# Resting State Functional Connectivity of Brain With Electroconvulsive Therapy in Depression: Meta-Analysis to Understand Its Mechanisms

**DOI:** 10.3389/fnhum.2020.616054

**Published:** 2021-01-21

**Authors:** Preeti Sinha, Himanshu Joshi, Dhruva Ithal

**Affiliations:** ^1^ECT Services, Noninvasive Brain Stimulation (NIBS) Team, Department of Psychiatry, Bengaluru, India; ^2^Geriatric Clinic and Services, Department of Psychiatry, National Institute of Mental Health and Neurosciences, Bengaluru, India; ^3^Multimodal Brain Image Analysis Laboratory, Department of Psychiatry, National Institute of Mental Health and Neurosciences, Bengaluru, India; ^4^Accelerated Program for Discovery in Brain Disorders, Department of Psychiatry, National Institute of Mental Health and Neurosciences, Bengaluru, India

**Keywords:** electroconvulsive therapy, depression, resting state functional neuroimaging, meta- analysis, activation likelihood estimation, hippocampus, dorsal anterior cingulate cortex, posterior cingulate cortex

## Abstract

**Introduction:** Electroconvulsive therapy (ECT) is a commonly used brain stimulation treatment for treatment-resistant or severe depression. This study was planned to find the effects of ECT on brain connectivity by conducting a systematic review and coordinate-based meta-analysis of the studies performing resting state fMRI (rsfMRI) in patients with depression receiving ECT.

**Methods:** We systematically searched the databases published up to July 31, 2020, for studies in patients having depression that compared resting-state functional connectivity (rsFC) before and after a course of pulse wave ECT. Meta-analysis was performed using the activation likelihood estimation method after extracting details about coordinates, voxel size, and method for correction of multiple comparisons corresponding to the significant clusters and the respective rsFC analysis measure with its method of extraction.

**Results:** Among 41 articles selected for full-text review, 31 articles were included in the systematic review. Among them, 13 articles were included in the meta-analysis, and a total of 73 foci of 21 experiments were examined using activation likelihood estimation in 10 sets. Using the cluster-level interference method, one voxel-wise analysis with the measure of amplitude of low frequency fluctuations and one seed-voxel analysis with the right hippocampus showed a significant reduction (*p* < 0.0001) in the left cingulate gyrus (dorsal anterior cingulate cortex) and a significant increase (*p* < 0.0001) in the right hippocampus with the right parahippocampal gyrus, respectively. Another analysis with the studies implementing network-wise (posterior default mode network: dorsomedial prefrontal cortex) resting state functional connectivity showed a significant increase (*p* < 0.001) in bilateral posterior cingulate cortex. There was considerable variability as well as a few key deficits in the preprocessing and analysis of the neuroimages and the reporting of results in the included studies. Due to lesser studies, we could not do further analysis to address the neuroimaging variability and subject-related differences.

**Conclusion:** The brain regions noted in this meta-analysis are reasonably specific and distinguished, and they had significant changes in resting state functional connectivity after a course of ECT for depression. More studies with better neuroimaging standards should be conducted in the future to confirm these results in different subgroups of depression and with varied aspects of ECT.

## Introduction

Electroconvulsive therapy (ECT) as a non-invasive brain stimulation treatment holds an important place in the management of depression (Hermida et al., 2018); it is more clinically and cost effective than other non-invasive brain stimulation methods in pharmacotherapy-resistant depression (Magnezi et al., 2016). The opening of different avenues of investigational modalities in the last 20 years has promoted a detailed examination of mechanisms of the effects of ECT vis-à-vis the neurobiology of depression to improve its applicability and tolerability (Li et al., 2020). Resting state functional magnetic resonance imaging (rsfMRI) is one such technique of neuroimaging, with which the spontaneous activities of the brain during rest are recorded through blood oxygen level dependent (BOLD) signals (Biswal et al., 1995). In comparison to stimulus-based acquisition protocols (task based) of fMRI, this is not only simpler, but it also can identify functionally and spatially distinct modes with greater biological interpretability (Fox and Raichle, 2007; Van Dijk et al., 2010). There are different analysis strategies/measures available for rsfMRI to understand the intrinsic functional connectivity at rest such as regional homogeneity (ReHo) (Zang et al., 2004), amplitude of low frequency fluctuations (ALFF) (Cordes et al., 2001) or fractional amplitude of low frequency fluctuations (fALFF) (Zou et al., 2008), resting state network based functional connectivity (FC) (Raichle et al., 2001; Beckmann et al., 2005), global measures-FC (Friston, 1994; Salvador et al., 2005), and graph theory and network (GTN) analysis (Bullmore and Sporns, 2009; Farahani et al., 2019). Under these strategies, many additional methodological approaches for extracting data are possible based on the research question, such as seed-voxel analysis, voxel-wise analysis, and local and global measures of network in graph theory (Smith, 2012). Each of them provides a window of opportunity to examine the FC of the brain noninvasively.

The rsfMRI studies using the abovementioned measures and methods and other kinds of neuroimaging studies have helped us to understand the disease mechanisms of depression. A recent meta-analysis on structural MRI–based studies in a depressed population established evidence of global atrophy of bilateral hippocampus (HC) (Santos et al., 2018). This finding adds to the integrated model of neurobiological, cognitive, and psychological construct toward the theory of neuronal loss and reduction of synaptic plasticity in HC and probably in the region of medial prefrontal cortex (PFC) in patients with depression (Price and Duman, 2020). Task-based fMRI in depression shows increased FC of the amygdala with HC and that of subgenual anterior cingulate cortex (sgACC) and insula and middle frontal gyrus with dorsal anterior cingulate cortex (dACC) during emotional/pain-related tasks (Helm et al., 2018). The sgACC could differentiate depressed patients from healthy controls in an rsfMRI study with both group-level clustering consistency and individual-level classification consistency of 92.5% (Zeng et al., 2014). In general, rsfMRI-based studies support the involvement of frontal, prefrontal, and limbic structures in depression with lesser consistency for a specific region (Helm et al., 2018). Other areas showing increased resting state FC (rsFC) in patients with depression include the right amygdala with ventral anterior putamen and reduced rsFC in middle occipital gyrus, inferior temporal gyrus, and retrosplenial cortex in the left hemisphere (Gray et al., 2020).

The neurobiological processes involved in the treatment of depression are said to be better understood with antidepressants than with different brain stimulation treatments and psychotherapy. Different antidepressants in task-based fMRI studies are found to normalize the increased activation of the amygdala and ACC, particularly sgACC, to negative emotional tasks and also to improve the activation of these regions to positive emotions (Arnone, 2019). In a few studies, antidepressants also reduced the activation in the dorsolateral PFC (dlPFC) with anticipatory cues/self-referential tasks and in the insula with negative emotions/pain-related tasks. The findings of rsfMRI studies, on the other hand, are too variable to give specific interpretations for the effects of antidepressants beyond the involvement of prefrontal and limbic structures and the default mode network (DMN) (Fonseka et al., 2018; Arnone, 2019). The neuroimaging predictors considered for response to antidepressants include HC, amygdala, ACC, posterior cingulate cortex (PCC), insula, orbitofrontal cortex, dlPFC, and dorsomedial prefrontal cortex (dmPFC); however, there is less consensus about the direction of change in these predictor regions (Fonseka et al., 2018).

ECT-associated structural changes in the brain in patients with depression are probably more reliable than similar changes with other treatments for depression (Enneking et al., 2020). These findings are also more consistent than the other neuroimaging modality–related findings in ECT. According to a recent systematic review, gray matter volume (GMV) of the amygdala, HC, and ACC increases in patients with depression following administration of ECT (Enneking et al., 2020). This review did not find these changes to be associated with the response to ECT. However, an earlier systematic review focusing only on limbic structures notes a negative association of the left HC GMV with a better clinical response to ECT (Takamiya et al., 2018). Similarly, another systematic review on baseline predictors reports that reduced GMV of HC and increased GMV of the amygdala and sgACC are predictive of a better ECT response in depression (Levy et al., 2019). Reviews are available that looked at changes in rsFC with ECT in depression. They include the other studies related to either other treatments for depression (Brakowski et al., 2017; Fonseka et al., 2018) or using other neuroimaging modalities with ECT (Abbott et al., 2014a; Bolwig, 2014; Zhuo and Yu, 2014; Yrondi et al., 2018). These reviews provide a broad notion about rsfMRI effects of ECT as altered FC in DMN, sgACC, central executive network, and dlPFC.

The above description of neuroimaging findings of depression and its various treatments, including ECT, is inferred from the narrative and systematic reviews. Another systematic approach of review, the meta-analysis, can correct the distorting effects of sampling error, measurement error, and other artifacts that produce the illusion of conflicting findings (Schmidt and Hunter, 2015). A meta-analysis may, thus, better integrate the findings across studies to reveal the specific patterns of relationships. This is particularly relevant in the fMRI field, considering the low power of individual studies and the variability present in scanning, preprocessing, and analysis of neuroimages in these studies (Samartsidis et al., 2017; Muller et al., 2018). With the development and progress in methods of coordinate based meta-analysis (CBMA) in the last 10 years, the meta-analytic approach of review has become increasingly common. Unlike image-based meta-analysis (IBMA), which requires the sharing of full image data, CBMA requires mainly information related to cluster size, its peak voxel coordinates, and related statistical methods used for analyzing neuroimages. Activation likelihood estimation (ALE), one of the most common methods described under CBMA (Samartsidis et al., 2017), has been utilized in meta-analysis for fMRI studies in depression. One such meta-analysis, focusing on the treatment of depression, finds a series of regions having altered FC with psychotherapy and activation in insula with antidepressants (Boccia et al., 2016), and a recent study using ALE on rsFC in all treatments for depression, including non-invasive brain stimulation, finds predictors for response to rTMS but not for ECT (Long et al., 2020). The major issues with both these papers and many other neuroimaging meta-analyses exploring the neurobiology of depressive disorders (Sacher et al., 2012; Kuhn and Gallinat, 2013) is they include the inappropriate combining of studies with different measures/extraction methods of rsfMRI (seed-based, ICA, ALFF, ReHo, FCD) (Zang et al., 2015) and inadequate qualitative analysis of the whole neuroimaging process of the included studies (Poldrack et al., 2008; Weber et al., 2015; Roiser et al., 2016; Soares et al., 2016).

Thus, we planned a systematic review and CBMA using ALE of the studies performing rsfMRI before and after a course of ECT in patients having depression. The primary objective of this meta-analysis was to provide definite and specific patterns of rsFC associated with ECT in depression by synthesizing the findings of different modalities of rsfMRI.

## Materials and Methods

### Search Strategies and Study Selection

In this systematic review, we follow the recommendations provided in the Preferred Reporting Items for Systematic reviews and Meta-Analysis (PRISMA) statement (Moher et al., 2009). A systematic literature search was performed in the following four electronic databases: PubMed, Medline, Pro Quest, and Web of Science library. The terms put together for this search are shown below:

- (electroconvulsive) OR (electroconvulsive therapy) OR (ECT) OR (Shock therapy)

AND

- (depression) OR (depressive disorder)

AND

- (resting state functional connectivity) OR (rsfMRI) OR (rs-fMRI) OR (bold rest) OR (rest fMRI) OR (functional connectivity at rest) OR (fMRI)

The search was conducted initially on April 1, 2020, and then it was repeated on July 31, 2020. A total of 477 studies were obtained, and they were then entered into the reference citation manager (Endnote X9) to remove the duplicates. Further selection of the articles was done on the basis of the following criteria.

#### Inclusion Criteria

1. Prospective observational/ randomized study
2. Subjects in the study having an episode of depression of any severity based on either DSM-IV/5 or ICD-10 irrespective of whether it is part of bipolar affective disorder or major depressive disorder, i.e., unipolar (single episode or recurrent depressive disorder)
3. Subjects in the study received constant current, pulse-wave modified ECT with any electrode placement and any set of electrical parameters
4. Subjects in the study at least underwent rsfMRI of brain on 2 occasions: (1) Prior to the beginning of the ECT course or (2) either at the end of the ECT course or after any fixed number of sessions.

There was no constraint in this review on the concurrent use of any medications for the treatment of depressive episodes.

#### Exclusion Criteria

1. Case reports or case series
2. Single ECT session
3. Simultaneous treatment with any other brain stimulation technique
4. Comorbid severe mental illness or neurological illness.

All 3 authors (PS, HJ, DI) of this manuscript reviewed the title and abstract of each article independently as per the abovementioned criteria. The studies that clearly satisfy the criteria or whose exclusion could not be confirmed based on review of the abstract, they were selected for acquisition of the full text. The final inclusion to the meta-analysis and systematic review was made after reviewing the full text. In case of any disagreement between any two reviewers during any step of the review, the third reviewer's decision was considered.

### Data Extraction

The data was extracted for each study under 2 major categories: subject characteristics and neuroimaging characteristics. The study was identified by its first author, journal, and year of publication. The details noted for the study sample included demography, psychiatry diagnosis along with specifically used clinical features as inclusion criteria (if present), pre- and post-ECT scores on rating scales used for depression, all standard ECT procedure–related information, and details of the concurrent psychotropics. Under the neuroimaging category, details about both scanning and preprocessing of rsfMRI, particulars of measures and methods of extraction undertaken to process the rsfMRI images, the statistical approaches adopted to analyze the differences between pre- and post-ECT neuroimages, and results of the analysis pertaining to rsfMRI were recorded.

Unlike the availability of tools for assessing the quality of general epidemiological, diagnostic, and intervention-related studies, we could not find any specific tool to rate the quality of fMRI aspects of a study. However, there are reviews (Waheed et al., 2016) and a few guidelines (Poldrack et al., 2008; Weber et al., 2015; Roiser et al., 2016; Soares et al., 2016) available focusing on how to conduct and report fMRI-based clinical studies. Based on these recommendations, we prepared a set of variables related to the process and analysis of rsfMRI as well as the display of results in the text, tables, and figures. There were a few articles for which the required information about the coordinates of peak voxel and cluster size of the significant regions were unavailable in their full text and Supplementary Material. We sought that information by writing to the respective corresponding authors.

### Activation Likelihood Estimation Analysis

CBMA of rsfMRI data was performed using a revised version of the ALE algorithm (Laird et al., 2005; Eickhoff et al., 2009, 2012; Turkeltaub et al., 2012) implemented in GingerALE 3.0.2. The consistency of the coordinates was assured using either Montreal Neuroimaging Institute (MNI) coordinates or converting them into an MNI-based coordinate system. Studies having a similar measure of rsFC were compiled together, followed by putting those results that had a change in one direction (either pre-ECT > post-ECT or post-ECT > pre-ECT) in one set for ALE analysis. The estimates thus obtained had the above-chance convergence of rsFC patterns, independently distributed, between common experiments/studies in a random effects model. The resulting ALE maps and ALE score corresponding to the experiments studied were extracted. Multiple comparisons were performed accordingly, either at a statistical (*p* < 0.05; 1,000 permutations) threshold using a cluster-level familywise error rate (FWER) or at a statistical threshold of *p* < 0.05 (minimum volume threshold in mm^3^ equal to the volume of the lowest cluster having significant difference) using the false discovery rate (FDR) as a correction measure.

## Results

### Search Results

**Figure 1:**
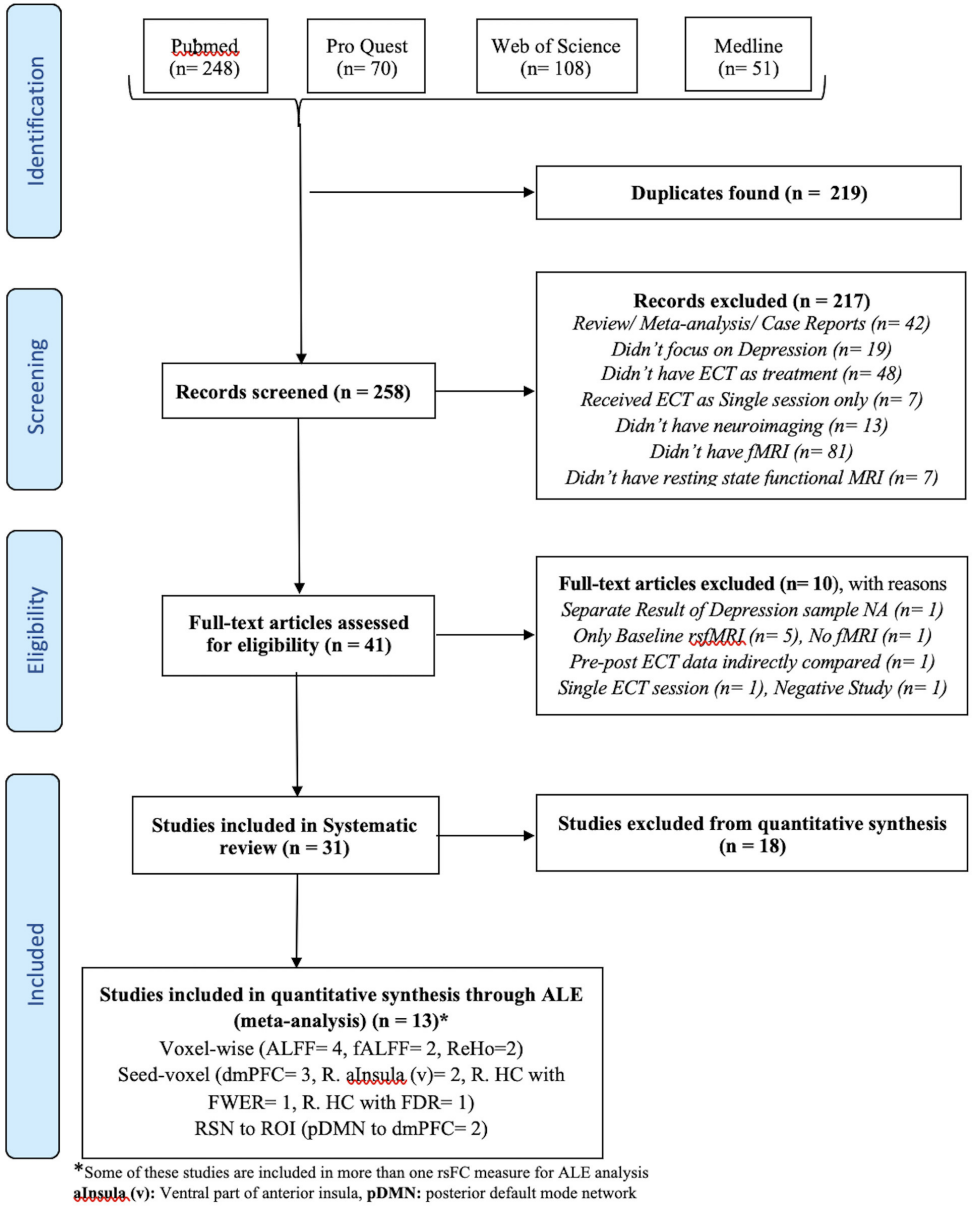
Flow diagram of literature search and study selection. *Some of these studies are included in more than one rsFC measure for ALE analysis. aInsula (v), Ventral part of anterior insula; pDMN, posterior default mode network.

The flow diagram of the process depicting the literature search and study selection is shown in Figure 1. Among the 258 articles found in the literature search, 217 were excluded after reviewing the abstract, and another 10 were rejected after reviewing their full text. Their details and reasons for exclusion from the systematic review are shown in Supplementary Table 1. We identified a total of 31 articles to be included in the systematic review. Among them, 13 articles were considered for ALE analysis. One of them had 2 different samples (Bai et al., 2018b), which were considered separately for meta-analysis. Contrary to it, there was one sample that was used for different kinds of neuroimaging analysis in different articles (Wang et al., 2017, 2018a,b; Wang J. et al., 2020; Wang L. et al., 2020). We considered those articles to be separate entities as the different measures of rsFC were undertaken separately for meta-analysis. The studies reporting ALFF, fALFF, and ReHo as a measure of rsFC with the voxel-wise method of extracting image data were analyzed using ALE. All of them used FWER for correcting multiple comparisons. Further, ALE was conducted for 3 seed-based FC. Here, the 2 studies reporting right HC (R. HC) as seed were considered separately during ALE because one used FWER (Abbott et al., 2014b) and the other used FDR (Takamiya et al., 2020) for handling multiple comparisons. Last, 2 studies reporting rsFC between the posterior DMN (constituting PCC and precuneus) and dmPFC using network-based analysis with FDR as the multiple comparisons correction method were included for ALE. The details of all these studies are presented in Table 1 with their measures that were included in ALE analysis as well as the excluded measures. The remaining 18 studies that were excluded completely from the meta-analysis are shown in Table 2 with all measures of rsFC used in these studies and reasons for their exclusion. The most common reason for the exclusion of studies was the absence of any other study using same measure or using same region of interest (ROI) for seed-/network-based rsFC analysis. In addition, some other studies were also not included due to the unavailability of information regarding coordinates and cluster size needed to conduct ALE analysis.

**Table 1:** List of studies with their different measures of rsFC included in ALE analysis examining the effect of ECT in depression.

**References**	**ALFF based rsFC**	**fALFF based rsFC**	**ReHo based rsFC**	**Seed based rsFC**	**Seed taken**	**RSN/ROI to RSN/ ROI**	**RSN taken**	**ROI taken**	**Other measures of rsFC**	**Correction for multiple comparisons**	**rsFC measure/ROI used in ALE analysis**	**Other studies included in ALE analysis of the respective rsFC measure/ROI**
Abbott et al. (2013)	-	-	-	-	-	RSN to RSN	*pDMN to dmPFC, L. dlPFC*	-	-	FDR	pDMN (PCC, Precuneus) with dmPFC	Leaver et al., 2016b
Abbott et al. (2014b)	-	-	-	Yes	*HC*	-	-	-	-	FWER	R. HC to R. TL	None
Liu et al. (2015)	Yes	-	-	Yes	L. sgACC	-	-	-	-	FWER	ALFF	Kong et al., 2017; Bai et al., 2018b
Argyelan et al. (2016)	-	Yes	-	Yes	SCC(peak coordinate on R. side)	-	-	-	-	FWER (fALFF); FDR (sbFC)	fALFF	Qiu et al., 2019
Leaver et al. (2016b)	-	-	-	-	-	RSNs to ROI	*mdTh/ vBGN, pDMN, aDMN, vDMN, SAL, OFN, AMTN*	*dACC, PCC, R. a TL, Precuneus, mdTh*,	GTN	FDR	pDMN (PCC, Precuneus) with dmPFC	Abbott et al., 2013
Qiu et al. (2016)	-	-	Yes	-	-	-	-	-	-	FWER	ReHo	Kong et al., 2017
Kong et al. (2017)	Yes	-	Yes	-	-	-	-	-	-	FWER	ALFF ReHo	Liu et al., 2015; Qiu et al., 2016; Bai et al., 2018b
Bai et al. (2018b)	Yes	-	-	Yes	*dmPFC*	-	-	-	-	FWER	ALFF Seed (dmPFC) to voxel	Liu et al., 2015; Qiu et al., 2016; Kong et al., 2017; Wang J. et al., 2020
Qiu et al. (2019)		Yes	-	-	-	-	-	-	-	FWER	fALFF	Argyelan et al., 2016
Wang L. et al. (2020)	-	-	-	Yes	*R. aInsula (v)*	-	-	-	-	FWER	Seed [R. aInsula (v)] to voxel	Zhang et al., 2020
Takamiya et al. (2020)	-	-	-	Yes	*R. HC, L. HC*	-	-	-	-	FDR	Seed (R. HC) to voxel	None
Wang J. et al. (2020)	-	-	-	Yes	*L. AG, dmPFC*	-	-	-	FcHo	FWER	Seed (dmPFC) to voxel	Bai et al., 2018b
Zhang et al. (2020)	-	-	-	Yes	*R. & L. aInsula (v)*	-	-	-	-	FWER	Seed [R. aInsula (v)] to voxel	Wang L. et al., 2020

*aDMN, anterior DMN; AMTN, Anteromedial Temporal network; mdTh, medio dorsal thalamus; OFN, Orbitofrontal network; pDMN, posterior DMN; SCC, Subcallosal cingulate cortex; SAL, Salience network; vBGN, ventral basal ganglia network; aTL, anterior Temporal lobe; GTN, Graph Theory and Network analysis; FcHo, Functional connectivity Homogeneity; FDR, False Discovery Rate; FWER, Family-wise Error Rate*.

**Table 2:** List of studies with their different measures of rsFC excluded from ALE analysis examining the effect of ECT in depression and the reasons for exclusion.

**References**	**Seed based rsFC**	**Seed taken**	**RSN/ROI to RSN/ ROI rsFC**	**RSN taken**	**ROI taken**	**Other measures of rsFC**	**Correction for multiple comparisons**	**Reason for exclusion from ALE analysis**
Beall et al. (2012)	-	-	ROI to ROI	-	*B/L ACC to OFC, Caudate*	-	Bonferroni	No other study has matching ROI to ROI in result, Coordinates information NA
Perrin et al. (2012)	-	-	-	-	-	weighted FC for voxel to voxel	FWER	No other study used same measure of rsFC analysis
Wei et al. (2014)	-	-	-	-	-	VMHC for voxel to voxel	FWER	No other study used same measure of rsFC analysis
Cano et al. (2016)	Yes	*R. cm/ sfAmyg*	-	-	-	-	FWER	No other study used same seed for Seed-voxel rsFC analysis
Leaver et al. (2016a)	-	-	RSN to ROI		*aDMN & vDMN to VS*	-	FWER	No other study has matching RSN to ROI in result
Mulders et al. (2016)	-	-	-	-	-	Variance ratio of FC within DMN mask for voxel to voxel	FDR	No other study used same measure of rsFC analysis
Wang et al. (2017)	Yes	*L. sfAmyg*					FWER	No other study used same seed for Seed-voxel rsFC analysis
Bai et al. (2018a)	Yes	*L. aHC, L. midHC*	-	-	-	-	FWER	No other study used same seed for Seed-voxel rsFC analysis
Wang et al. (2018a)	-	-	RSN to RSN, ROI to ROI	Details in Table 5	Details in Table 5	-	Bonferroni	No other study has matching RSN/ROI in result, Coordinates information NA
Wang et al. (2018b)	Yes	*R. supTG*	-	-	-	FCD	FWER	No other study used same measure of rsFC or used same seed for Seed-voxel rsFC analysis
Wei et al. (2018)	-	-	-	-	-	Person's correlation of FC for voxel to voxel	FWER	No other study used same measure of rsFC analysis
Li et al. (2019)	-	-	-	-	-	gFCD	FWER	No other study used same measure of rsFC analysis
Sinha et al. (2019)	-	-	-	-	-	GTN	FDR	GTN is not analyzed with ALE
Leaver et al. (2020)	-	-	-	-	-	GTN within HC	FDR	GTN is not analyzed with ALE
Qi et al. (2020)	-	-	-	-	-	GMV (sMRI) + fALFF (rsfMRI)	FDR	Guidelines for combining different modality of MRI in meta-analysis: NA
Sun et al. (2020)	-	-	ROI to ROI	-	Details in Table 5	-	FDR	No other study has matching ROI to ROI in result, Coordinates information NA
Wei et al. (2020b)	Yes (within cerebellum)	*L. sgACC*	-	-	-	-	FWER	No other study used similar mask
Wei et al. (2020a)	Within Thalamus	*Parietal Cortex, L. Pulvinar*	-	-	-	-	FWER	No other study used same seed for Seed-voxel rsFC analysis

*ACC, Anterior Cingulate Cortex; OFC, Orbito Frontal Cortex; aDMN, anterior DMN; cm/sfAmyg, centromedian/superficial amygdala; midHC, middle hippocampus; supTG, superior temporal gyrus; vDMN, ventral DMN; VMHC, Voxel-Mirrored Homotopic Connectivity; VS, Ventral striatum; sgACC, subgenual Anterior Cingulate Cortex; gFCD, global Functional Connectivity Density; FDR, False Discovery Rate; FWER, Family-wise Error Rate*.

### ALE Results

**Figure 2:**
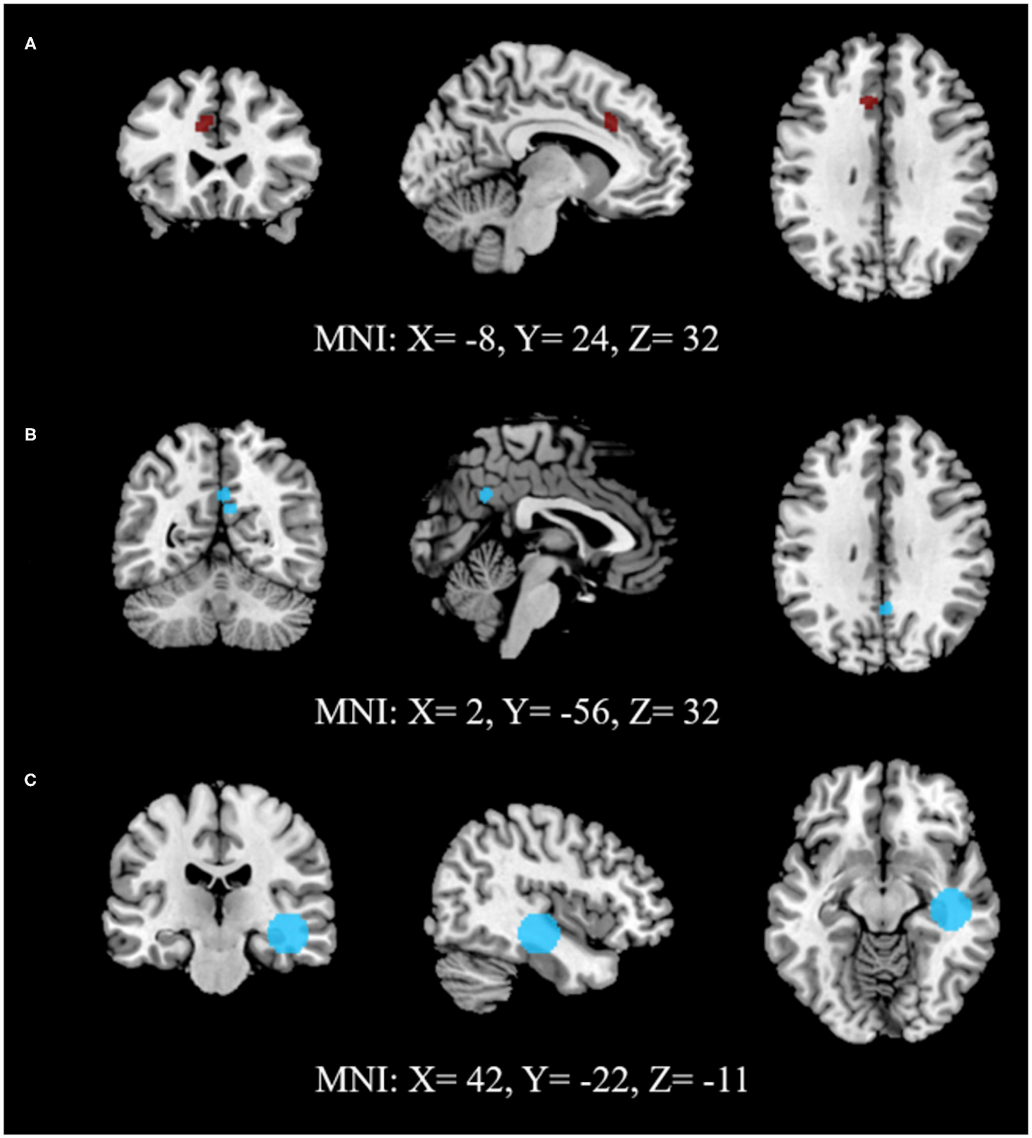
Activation likelihood estimate results for studies measuring **(A)** ALFF, **(B)** network connectivity and **(C)** seed (RHC) to voxels to study the effect of ECT on rsFC. **(A)** shows lower (dark red) and **(B,C)** show higher (light blue) rsFC after ECT intervention. **(A,C)** use FWE (cluster-level extent thresholding) unlike **(B)**, which used FDR as correction for multiple comparisons during reporting significant changes between pre- and post-intervention (Refer to Table 3 for details).

A total of 73 foci were analyzed from 21 experiments through 10 sets of ALE analysis. Among the analysis exploring ECT-associated rsFC changes using FWER, one voxel-wise analysis with the measure of ALFF and one seed-voxel analysis with R. HC showed a significant reduction of rsFC (*p* < 0.0001) in the left cingulate gyrus (L. CG) in the area of L. dACC and a significant increase (*p* < 0.0001) in R. HC with right parahippocampal gyrus (R. PHG) using cluster-level interference method, respectively. Another combination of studies implementing network-based (posterior DMN- dmPFC) rsFC analysis using FDR as a multiple comparisons correction procedure showed a statistically significant increase (*p* < 0.001) in the left posterior cingulate cortex (L. PCC) and R. PCC using FDR. The results are presented in Table 3, and images of significant clusters are shown in Figure 2.

**Table 3:** ALE results for studies using different measures of rsFC to study the effect of ECT in depression.

**Analysis**	**ECT**	**FDR/ FWER**	**Brain region**	**Cluster size (mm^**3**^)**	**Peak MNI Coordinates (x, y, z)**	**ALE value**	***Z* score**	***P***
ALFF voxel wise	Pre > post	FWER^*^	L. CG, BA 32	584	−8, 24, 32	0.011	4.715	<0.0001
ALFF voxel wise	Post > pre	FWER^*^	No significant clusters found					
ReHo voxel cluster	Pre > post	FWER^*^	No significant clusters found					
Network Based	Post > pre	FDR	L. PCC, BA 31 R. PCC, BA 31	600	2, −56,32 8, −56, 24	0.014 0.013	5.378 5.165	<0.001 <0.001
fALFF voxel to voxel	Pre > post	FWER	No significant clusters found					
Seed (R. HC) to voxel	Post > pre	FWER^*^	R. HC and R. PHG	8864	42, −22, −11	0.008	4.296	<0.0001
Seed (R. HC) to voxel	Post > pre	FDR	No significant clusters found					
Seed (R. HC) to voxel	Pre > post	FDR	No significant clusters found					
Seed (dmPFC) to voxel	Post > pre	FWER^*^	No significant clusters found					
Seed (L. anterior Insula (ventral) to voxel	Post > pre	FWER^#^	No significant clusters found					

* Cluster level FWER;

# *Voxel level FWER*.

*pre> post, Greater before the ECT course; post> pre, Greater after the ECT course/specified number of ECT sessions*.

### Articles Included in Meta-Analysis-Quality Assessment of Neuroimaging Process

The detailed qualitative assessment of neuroimaging was done for those studies that were included in ALE analysis (Supplementary Table 2). We did not rate the overall neuroimaging quality of a study. Instead, we highlighted the aspects by which the study lacked the relevant information or missed the concerned step of the neuroimaging process. These lacunae definitely bring down the quality of studies.

#### Scanning Procedure

- Most of the studies provided almost all relevant information about it although some did not document a few characteristics, such as orientation of image acquisition, matrix size, and the presence of interslice skip.
- Although many articles provided the name of the software/s used for preprocessing and further analysis of images, detail about the version was missing in most of them.

#### Image Preprocessing

- The information about the distortion correction related to the artifacts of the EPI sequence was available only in Leaver et al. (2016b). In this study, artifacts retrieved through ICA were then used as regressors during denoising to get rid of them, an approach suggested for handling the distortions (Griffanti et al., 2016; Soares et al., 2016). None of the other papers reported this or any other method of correction, such as reversed phase encoding, field map correction, or point spread function (Hong et al., 2015; Caballero-Gaudes and Reynolds, 2017; Nunes and Hajnal, 2018).
- All studies except Argyelan et al. (2016) provided information about realignment parameters for head motion correction, but a description about transformation functions used during realignment was specified only in Abbott et al. (2013); Abbott et al. (2014b).
- Outlier detection was performed through framewise displacement (FD) in only 4 studies (Argyelan et al., 2016; Bai et al., 2018b; Takamiya et al., 2020; Wang J. et al., 2020) with the threshold as 0.5 or 5 mm. Other studies did not give any account of outlier detection.
- The normalization of the functional images was indirect through the structural image and its associated template in 3 articles (Liu et al., 2015; Kong et al., 2017; Zhang et al., 2020). In other studies, the normalization was probably direct. The name of the template with or without further specification was provided by all except Bai et al. (2018b). An EPI template was used only by Qiu et al. (2016), whereas others used a standard structural template.
- FWHM for smoothing varied from 3.33 times the slice thickness (Abbott et al., 2013, 2014b) to <1.33 times (Qiu et al., 2016; Kong et al., 2017; Bai et al., 2018b; Zhang et al., 2020). They had smoothing with FWHM at 3 times (Leaver et al., 2016b), 2.67 times (Qiu et al., 2019), 2 times (Argyelan et al., 2016), and 1.5 times (Wang J. et al., 2020; Wang L. et al., 2020).
- Some of the recommended models/measures for denoising were reported in few studies here. tCompCor was adopted by Abbott et al. (2013, 2014b) and aCompCor by Takamiya et al. (2020), and FD-related motion parameters were scrubbed by Takamiya et al. (2020) and Wang J. et al. (2020). Nonetheless, cerebrospinal fluid, white matter, and motion parameters from realignment were considered by all during linear regression for denoising along with the frequency band filtering. Leaver et al. (2016b) did not use these parameters for denoising providing statistical justification, and Takamiya et al. (2020) did not provide information about the number of motion parameters used during denoising. No study recorded physiological parameters specifically to be used as regressors. The global brain signal was used as a regressor by Liu et al. (2015) and Zhang et al. (2020), whereas a few studies did not consider it (Abbott et al., 2013, 2014b; Argyelan et al., 2016; Bai et al., 2018b; Wang J. et al., 2020; Wang L. et al., 2020). No study provided any information about detrending. There were two studies that did not have any information about denoising (Qiu et al., 2016, 2019).

#### Statistical Analysis

Studies had a few shortfalls in this area compared with the standards required (Poldrack et al., 2008; Roiser et al., 2016).

- Although most of them used FWER for handling multiple comparison issues, a few did not write about the model used to consider the cluster size and significance threshold for FWER (Abbott et al., 2014b; Argyelan et al., 2016; Zhang et al., 2020).
- Among the studies that used random field theory for FWER, none of them provided the information about resolution element (RESEL) count (Leaver et al., 2016b; Kong et al., 2017; Bai et al., 2018b). A RESEL is defined as a block of pixels of the same size as the FWHM of the smoothness of the image and is a crucial factor in the application of random field theory for FWER (Brett et al., 2003).
- In some studies, the difference between pre- and post-ECT rsfMRI was analyzed without adding any study sample-based characteristic as a covariate (Liu et al., 2015; Argyelan et al., 2016; Kong et al., 2017; Wang J. et al., 2020; Wang L. et al., 2020).
- The correlation of ECT-associated rsFC with the clinical characteristics was carried out in most of the studies as a *post hoc* analysis of the significant results without any correction for multiple comparisons. In one study only, we found the percentage change in depression scores during the course of ECT to be incorporated as between-subjects contrast in the primary model of analysis of significant change in seed-based rsFC (Takamiya et al., 2020).
- The display of the results in tabular format was mostly as per the standards in all the studies included for meta-analysis although some had a deficiency in a few aspects in their figures, such as absence of thresholds, *t-*scores, naming, and coordinate details of significant regions. In addition, there were some studies that could not be included in the meta-analysis due to unavailability of all required parameters of the significant results.

### Articles Included for Systematic Review: Clinical and ECT Characteristics

#### Articles Included in Meta-Analysis: Clinical Characteristics

The clinical characteristics were reported adequately by all studies. Among the 13 studies (Table 4) included in the meta-analysis, the sample size was limited to 12–30 subjects except for one study by Zhang et al. (2020) with 45 subjects. Half of these studies had patients with unipolar depression only, whereas the rest included subjects with bipolar depression as well. Three studies focused only on patients having treatment-resistant depression (Abbott et al., 2013; Argyelan et al., 2016; Leaver et al., 2016b), and four had older adults only as their participants (Abbott et al., 2013, 2014b; Kong et al., 2017; Takamiya et al., 2020). Except for a few (Argyelan et al., 2016; Leaver et al., 2016b; Qiu et al., 2016, 2019), patients in all studies had pharmacotherapy concurrently during the course of ECT.

**Table 4:** List of studies included in ALE analysis: clinical characteristics.

**1st Author (year)**	**Sample characteristics**	**ECT characteristics**
	**•Total No., Age as Mean (SD), M: F •Disease details •Medication details (Class, Frequency in %) •Depression scale [Name: Pre ECT score Mean (SD), Post ECT score Mean (SD)]**	**•Pulse width, electrode placement, charge as times ST •Frequency of ECT session, session with Post ECT MRI- [Fixed no/ Last session as Mean (SD)] •Anesthetic (Name, Dose), Muscle relaxant (Name, Dose)**
Abbott et al. (2013)	•12, 66.42 (9.78), 4:8 •Treatment resistance depression •AD (100%), AP (66.67%), MS (16.67%) •HAM-D: 34.56 (10.03), 2.89 (2.93) s	•Brief, RUL (10) & BT (2), 6 times (RUL) 2 times (BT) •3 times a week, > 5 days (21.13 ± 13.90) •after Last session- 11.17 (3.33) •Methohexital, S Ch
Abbott et al. (2014b)	•19, 65.3 (8.0), 6:13 •UPD •AD (94.7%), AP (63.2%) •HAM-D: 32.6 (8.5), 8.4 (8.6)	•**NA**, RUL (17) & BT (2), 6 times (RUL) 2 times (BT) •3 times a week, > 5 days (11 ± 8.4) after Last session- 11 (2.7) •Methohexital, S Ch
Liu et al. (2015)	•23, 30.57 (9.43), 9:1z •UPD with Active suicidal risk •1 AD (68.2%), 2 AD (27.3%), AP (13.6%); SSRI (68.2%), SNRI (18.2%), NaSSA (36.4%) •HAM-D: 28.45 (4.93), 8.23 (4.55)	•Brief, BT, 1.5–2 •1st 3 daily, then 3times a week, After 8th session •Propofol (1.5–2 mg/kg), S. Ch (0.5–1 mg/kg)
Argyelan et al. (2016)	•16, 48.5 (13. 6), 10:6 •Treatment resistance Depression (UPD = 13, BPD = 3) •All medications stopped except lorazepam •HAM-D: 28.2 (5.6), 10.3	•Brief, BF, 1.5 •3 times a week, Last or 8th session- 6.4 (1.5) •Ketamine (1mg/kg)/ Methohexital 1mg/kg, S Ch. 1mg/kg
Leaver et al. (2016b)	•30, 40.90 (12.45), 16:14 •Treatment resistance Depression (UPD = 24, BPD = 6) •Medications stopped 48–72 h prior to ECT course •HAM-D: 26.3 (5.8), 9.3 (5.5)	•**NA**, RUL, 5 times •3 times a week, Last session- 10.04 (2.93) •Short acting anesthetic, **NA**
Qiu et al. (2016)	•12, 34.4 (10.1), 4:8 •UPD •No medications •HAM-D: 35.9 ± 1.3, **NA**	•Brief, BT, **NA** •1st 2weeks as 3times a week, then twice in 1 week; After 8th session •Thiopentone (3.0–5.0 mg/kg) and S. Ch (0.5–1.0 mg/kg)
Kong et al. (2017)	•13, 63.0 (4.9), 2:11 •Severe depressive episode without psychotic symptoms •1 AD (53.8%), 2 AD (46.2%), AP = 0, MS = 0; SSRI (92.3%), NaSSA (46.2%), SNRI (7.7%) •HAM-D: 38.6(3.3), 3.1(2.9)	•Brief, BF, **NA** •3 times a week, Last session: 5.8(0.4) •Propofol, S. Ch
Bai et al. (2018b) (AMHU)	•33, 35.97 (11.11), 15:18 •UPD = 25, BPD = 8 •SSRI (66.7%), SNRI (33.3%), NaSSA (6.1%), SARI (6.1%), AP (51.5%); AC (Stopped) •HAM-D: 22.42 (4.12), 5.24 (5.09)	•**NA**, BF, **NA** •3 times a week, Last session- 8.03 (1.91) •Propofol, S. Ch
Bai et al. (2018b) (USTC)	•28, 35.25 (11.48), 6:22 •UPD = 23, BPD = 5 •SSRI (64.3%), SNRI (39.3%), NaSSA (28.6%), SARI (7.1%), AP (46.4%); AC (Stopped) •HAM-D: 21.54 (4.73), 8.36 (5.62)	•**NA**, BF, **NA** •3 times a week, Last session- 8.71 (1.80) •Propofol, S. Ch
Qiu et al. (2019)	•24, 31.33 (10.79), 10:14 •Severe UPD •No medication in last 1 month and during the ECT course •HAM-D: 31.33 (4.55), 8.58 (5.62) s	•Brief, BT, 1.5–2 times •1st 2 weeks as 3 times a week & 2 times a week in 3rd week, After 8th session •Thiopentone 3–5 mg/kg, S Ch (0.5–1mg/kg)
Wang L. et al. (2020)	•23, 38.74 (11.02), 11:12 •UPD (Treatment resistance or for suicide) •1 AD (86.9%), 2 AD (13.1%), AP (39.1%); SSRI (82.3%), SNRI (21.7%), NaSSa/ SARI (8.6%) •HAM-D: 22.22 (4.74), 3.83 (2.15)	•**NA**, BF, **NA** •1st 3 daily, then 3 times a week; Last session- 7.36 (2) •Propofol, S Ch
Takamiya et al. (2020)	•27, 67.5 (8.1), 8:19 •Depression with melancholic features (UPD = 22, BPD = 5) •AD (88.9%), AP (77.8%), MS (7.4%) •HAM-D: 32.0 (6.6), 6.0 (5.3)	•Brief, BL, **NA** •2-3 times a week, Last session: 10.8 (1.8) •Propofol (1 mg/kg), S. Ch (0.5–1 mg/kg)
Wang J. et al. (2020)	•23, 38.74 (11.02), 11:12 •UPD (Treatment resistance/ suicide) •1 AD (86.9%), 2 AD (13.1%), AP (39.1%); SSRI (82.6%), SNRI (21.7%), NaSSA (4.34%) •HAM-D: 22.22 (4.74), 3.83 (2.15)	•**NA**, BF, **NA** •1st 3 daily, then 3 times a week; Last session: 7.26 (2) •Propofol, S. Ch
Zhang et al. (2020)	•45, 39.07(12.29), 11:34 •UPD = 36, BPD = 9 •SSRI (62.22%), SNRI (31.11%), AP (55.55%) •HAM-D: 24.11(5.63), **NA**	•Brief, BF, **NA** •1st 3 daily, then 3times a week, Last Session: Range (6–12) •Propofol (0.2–0.5 mg/kg), S. Ch (0.5–1 mg/kg)

*AD, Antidepressants; AMHU, Anhui Mental Health Center as Study site; AP, Antipsychotics; BPD, Bipolar depression; BT, Bitemporal; HAM-D, Hamilton Depression Rating Scale; MS, Mood Stabilizer; NA, Information Not Available; NaSSA, Noradrenergic and specific serotonergic antidepressants; SARI, Serotonin antagonist and reuptake inhibitor; S. Ch, Succinylcholine; SD, Standard Deviation; SNRI, Serotonin-norepinephrine reuptake inhibitor; SSRI, Selective serotonin reuptake inhibitor; UPD, Unipolar depression; USTC, University of Science and Technology of China as Study site*.

#### Articles Excluded From Meta-Analysis: Clinical Characteristics

The clinical characteristics of the 18 articles that were excluded from meta-analysis were partially similar to those of articles included in the meta-analysis. Among these studies (Supplementary Table 4), all had a sample size of <30 subjects except for 3 studies with sample sizes of 45 (Bai et al., 2018a), 118 (Qi et al., 2020), and 122 (Sun et al., 2020), respectively. Ten of them recruited patients with unipolar depression only, and six of them solely focused on treatment-resistant depression. However, none of these studies specifically studied the geriatric population.

#### Articles Included for Systematic Review: ECT Characteristics

The treatment aspects related to ECT are presented here together for the articles included in the meta-analysis and those excluded. The details about ECT were provided sufficiently in most of the 31 articles for systematic review. However, the ratio of the administered electrical charge to the seizure threshold is mentioned only in some studies. This information is important as the electrical stimulus dosing influences the rate of improvement and total response in depressive symptoms with ECT (Murugesan, 1994). A few studies also fail to provide anesthetic medications details. An almost equal number of studies used bifrontal (BF), bitemporal (BT), and right unilateral (RUL) as electrode placements during ECT. Some had provision to switch to BT (Leaver et al., 2020; Qi et al., 2020; Sun et al., 2020) or BF (Leaver et al., 2016b) if RUL did not provide significant improvement. Except for Leaver et al. (2020) (ultra-brief pulse-wave ECT), all studies used brief pulse-wave ECT. Most of them had conducted post-ECT neuroimaging after the last session of ECT except in some studies, in which it was done after a predetermined number of ECT sessions (Liu et al., 2015; Cano et al., 2016; Qiu et al., 2016, 2019; Li et al., 2019; Sinha et al., 2019).

#### Articles Included for Systematic Review: Neuroimaging Findings

Here, we briefly present the resting state neuroimaging findings of all 31 articles that studied ECT-associated changes in rsFC in the depressed patient group. The detailed findings are shown in Supplementary Tables 3, 4. Among studies based on the voxel-wise method of data analysis, CG was often noted as a significant region to be associated with ECT. Although the ACC (L > R) is a more common region of the CG to show a significant change in rsFC after ECT, the concurrence was low for the specific part of the ACC. The other regions that had significant findings post-ECT in voxel-wise analysis of rsFC belonged to the frontal cortex and parietal cortex as well as the temporal cortex. It included dmPFC, bilateral precentral gyrus, bilateral superior frontal gyrus, left angular gyrus, left precuneus, bilateral HC, right superior temporal gyrus, and right insula. In addition, the cerebellum (L > R) in a few studies showed significant change in rsFC with ECT.

In seed-based analysis, rsFC of the sgACC/subcallosal cingulate cortex with ipsilateral PHG and contralateral temporal pole significantly changed with ECT in two studies but had contrast in the direction of change (Liu et al., 2015; Argyelan et al., 2016). Superficial amygdala was used in two studies for seed-based rsFC analysis. One study found a significant decrease in rsFC post-ECT between the centromedial/superficial amygdala and sgACC on the right side (Cano et al., 2016) although the other study noted an increase in rsFC of the superficial amygdala with a fusiform area on the left side (Wang et al., 2017). In network-based and ROI (with rsFC) to ROI analysis, ACC, PCC, different regions of DMN (PCC, precuneus, medial PFC, intraparietal sulcus), and left cerebellum more often had prominent changes in rsFC with ECT. In the study that jointly analyzed structural MRI and rsfMRI images using HAM-D scores as a reference, FC in PFC, HC, insula, and left caudate were found to be reduced after ECT (Qi et al., 2020).

## Discussion

Advancement in neuroimaging in the last 20 years has been seen as a hope to reduce the enigma associated with mechanisms of actions of ECT. Structural neuroimaging shows stronger evidence of change in the brain with ECT compared with other treatments for depression (Enneking et al., 2020). We focus on CBMA of rsfMRI-based studies conducted on patients receiving ECT for treating depression. A meta-analytic approach helped us to achieve reliable and strong results instead of a gamut of less reproducible findings of the individual studies. We conducted ALE analysis on 7 measures of rsFC, including ALFF, fALFF, and ReHo for the voxel-wise, ventral part of the anterior insula, dmPFC and R. HC as seed-based and pDMN-dmPFC as network-based data extraction methods from the rsfMRI data.

### Findings of Meta-Analysis and Systematic Review

The significant regions in our meta-analysis were associated with cingulate gyrus (L > R) and included the dorsal part of the ACC (Left), BA 32, and PCC (bilateral), BA 31. There was a reduction in rsFC of the L. dACC after the course of ECT. Neuroimaging studies suggest increased activity in the ACC as an important biomarker for depression (Helm et al., 2018; Lai, 2019), which normalizes after treatment with antidepressants and serves as a predictor for the response (Arnone, 2019; Dunlop et al., 2019; Tian et al., 2020). However, sgACC/rACC are implicated here more often than dACC. Studies exploring dACC found increased FC of dACC within the frontocingular network during emotional/cognitive control–related tasks in patients with depression (Schlösser et al., 2008), which also predicts response to both antidepressants (Crane et al., 2017; Godlewska et al., 2018) and psychotherapy (Beevers et al., 2015; Fonseka et al., 2018). Although Fu et al. (2004) found a decrease in FC of dACC with fluoxetine during the task of “sadness” recognition, most of the other studies on rsFC or task-based FC failed to observe a change in FC of dACC with the treatment of depression through either antidepressants or psychotherapy. Thus, the effect on the dACC in patients having depression, as noted in our meta-analysis, may be a specific mechanism of action of ECT. The dACC is implicated in the salience network along with the anterior insula (Seeley et al., 2007; Enneking et al., 2020). In fact, the dACC is being considered as a part of the neural alarm system and seems to be involved in both detecting performance in a cognitive task and social behavior as well as providing a negative affect to, thus, perceive errors and social rejection, respectively (Spunt et al., 2012). The exaggerated pattern of this aspect is associated with depression (Slavich et al., 2010; Kupferberg et al., 2016).

In our pDMN- and dmPFC-related network analysis, we found increased rsFC of PCC by the end of the ECT course. PCC is considered to be an important part of pDMN and is found to have increased FC with dmPFC in people having depression compared with controls (Mulders et al., 2015; Helm et al., 2018). Increased rsFC of PCC has been shown to be a predictor for response to antidepressants (Goldstein-Piekarski et al., 2018; Dunlop et al., 2019), psychotherapy (Dunlop et al., 2019), and ECT (van Waarde et al., 2015). Although there is evidence of reduction in FC of PCC with antidepressants in response to a negative emotional task in some studies, the evidence is limited in rsFC studies to overall increased activity in pDMN rather than in PCC specifically (Arnone, 2019; Ichikawa et al., 2020). An increase in glucose metabolism in PCC in unipolar depressed patients receiving fluoxetine was, however, noted in an earlier PET study (Mayberg et al., 2000). The possible reasons for the difference in brain regions affected by ECT and antidepressants may be related to their duration of action. The reduction in depressive symptoms achieved by the antidepressants is not by their direct pharmacological actions but through the brain's compensatory responses to those actions, hence, needing a longer time for the clinical improvement with antidepressants (Schatzberg and DeBattista, 2019). Considering that response to ECT is faster, its mechanism of action might be different from that of antidepressants.

The link of global atrophy of HC to the pathology of depression is reasonably recognized, and so is the improvement in its size and associated neurogenesis with antidepressants and other treatment for depression (Helm et al., 2018; Santos et al., 2018; Lai, 2019; Price and Duman, 2020). However, the knowledge about FC of HC during the depressive episode and post response to antidepressants or ECT is ambiguous (Fonseka et al., 2018; Dunlop et al., 2019). Hence, our result of increased FC between R. HC and R. PHG after a course of ECT is valuable. HC is considered to be part of the limbic system with PHG and amygdala and is involved in emotional perception, forming an integral part of the frontolimbic network (Yeo et al., 2011; Lindquist et al., 2012). Our result is based on a single-study ALE analysis using FWER; ALE analysis with multiple studies is definitely needed to confirm this finding and to examine the connectivity of HC of each hemisphere with prefrontal areas. There is also a need for a greater number of studies to evaluate the effect of ECT on other important areas that were noted during systematically reviewing existing studies. These possible regions include sgACC, dlPFC, precuneus, precentral gyrus, superior frontal gyrus, superior temporal gyrus, and anterior insula.

### Strengths of Our Meta-Analytic Approach

We analyzed the studies with different rsFC measures/extraction methods of rsfMRI separately for CBMA as recommended (Zhang et al., 2015). In fact, the studies having different seed regions in seed-voxel or RSN analysis were also analyzed separately. Combining seed-based connectivity studies with different seeds can be a problem because it represents selection bias at the time of choosing seeds and, hence, is not recommended (Cortese et al., 2020). This approach is distinct from that considered by earlier studies using gingerALE based meta-analysis, in which they had combined results of different kinds of neuroimaging (Chen et al., 2017; Disner et al., 2018; Mothersill and Donohoe, 2019), different approaches to fMRI (resting state and task based) (Ayoub et al., 2018), different extraction methods and measures for rsFC (Disner et al., 2018; Gu and Zhang, 2019; Lau et al., 2019), and different seed regions and networks (Lau et al., 2019; Ramsay, 2019; Xu et al., 2019). We also analyzed the studies separately that derived results using FDR and that using FWER as these statistical methods of correcting for multiple comparisons are fundamentally distinguished and need different handling during ALE analysis (Roiser et al., 2016; Eickhoff et al., 2017). Our results can be trusted with a greater degree of confidence considering that our *p* < 0.0001. Because foci with only the same direction of change was considered together in our analysis, our results could indicate significant regions with precision and the associated direction of effects with ECT unlike other neuroimaging meta-analyses using the ALE method (Mothersill and Donohoe, 2019; Gray et al., 2020).

### Limitations of the Studies Included in Meta-Analysis

#### Limitations in Clinical Characteristics

The most important limitation of the studies included in the meta-analysis is their small sample size. Half of the studies had a sample size fewer than 20, and the remainder of the studies except one (Wang L. et al., 2020) had sizes within 30. Small sample-sized studies have limited power and are more likely to miss the regions with significant FC or else to get spurious results if less stringent cutoff *p* value or lower voxel/cluster thresholds are used (Carter et al., 2016). The clinical population varied across the studies with inclusion of different categories of depression (unipolar vs. bipolar, with or without psychotic symptoms, presence of treatment resistance), age groups of both young and older adults, and varied status of pharmacotherapy. Studies also varied with the electrode placement used for administering ECT. All this variability in clinical and treatment characteristics might have added to the disparity in study findings and, hence, to the insignificant results in ALE analysis.

#### Limitations in Neuroimaging Characteristics

In addition, there were differences in acquisition and analysis of neuroimaging in the included studies. Because there is no scale/instrument available that rates the neuroimaging aspects of studies, many meta-analysis-based papers either had not commented on the quality of neuroimaging (Disner et al., 2018; Gu and Zhang, 2019; Ramsay, 2019) or did partly (Chen et al., 2017; Ayoub et al., 2018; Xu et al., 2019). We reviewed in detail the procedure, preprocessing, and analysis of neuroimaging; their documentation; and the reporting style of the results presented in the studies to assess the quality and understand the variability. Many features in the included studies were present as per the recommendations and opinions of experts (Poldrack et al., 2008; Weber et al., 2015; Soares et al., 2016), yet they had a few important omissions. Along with flip angle during scanning, the slice thickness varied; both would affect the image intensity. A few recommended steps of preprocessing were missing in many studies, thus reducing the validity of the respective neuroimaging study findings. These included a specific distortion correction method for scanner-related artifacts, outlier detection through DVARS (the temporal derivative of time courses for FC variance over voxels)/FD for further motion correction, and denoising with extensive variables and scrubbing using appropriate functions (aCompCor, ICA based) (Behzadi et al., 2007; Poldrack et al., 2011; Power et al., 2015; Griffanti et al., 2016; Caballero-Gaudes and Reynolds, 2017). We could not ascertain whether the unreported steps were carried out as many studies did not provide the version of software used for neuroimage preprocessing and analysis. The inadequate information provided about FWER in some studies further casts a concern about the accuracy of their results (Poldrack et al., 2008; Weber et al., 2015; Soares et al., 2016). Last, most of the studies correlated results with depressive symptoms as *post hoc* analysis, which increases the type-1 error (Vul et al., 2009).

### Limitations of Our Meta-Analytic Approach

Our study has few limitations as well. CBMA applied in our study has disadvantages, including less consistency and reliability of findings and less flexibility than IBMA, which relies on statistical parametric maps of raw images of the included studies (Salimi-Khorshidi et al., 2009). In addition, the role of the different demographic and clinical characteristics of study samples as covariates in explaining the significant results is still in its nascent phase in CBMA (Tench et al., 2020). This came as an important drawback for our analysis as we had significant heterogeneity in the included studies. However, using CBMA enabled us to include more studies than what was possible with image-based meta-analysis.

Among the available kernel-based techniques of CBMA [multilevel kernel density analysis, ALE, and signed differential mapping (SDM)], ALE is the most widely used and popular method (The BrainMap Project, 2020). With recent updates, ALE addresses the limitations cited with respect to multilevel kernel density analysis (Wager et al., 2007). The newer version of SDM as a seed-based d mapping permutation of subject imaging (SDM-PSI) is able to provide a good estimate of effect size of voxel clusters with a significant change in activity if the peak coordinates and *t*-values are reported (Albajes-Eizagirre et al., 2019). Unfortunately, many studies do not report *t-*values or associated *z*-values, and SDM-PSI is less sensitive and has more uncertainty than anisotropic effect-size seed-based d mapping (AES-SDM) (Radua et al., 2012). Other limitations exist with SDM-PSI, some of which are related to the principle of CBMA. These include the handling of studies using multiple comparisons, the presence of a fewer number of studies, and focusing on correlation among only those voxels that are completely in line with each other rather than partly. A recent meta-analysis of task-based fMRI for language comprehension in children found the same brain regions of significant activation peaks with both ALE and SDM-PSI (Enge et al., 2020). We applied the ALE as recommended and avoided mixing of the studies with differences in neuroimaging techniques and analysis (Zang et al., 2015).

We did not explore the data using model-based methods, such as the Bayesian hierarchical cluster process model, which could have provided more accurate spatial results (Kang et al., 2011). We hope that our results and future studies would lead to model-based CBMA of rsFC in ECT with a valid a priori assumption (Samartsidis et al., 2017). We also had to exclude studies using GTN due to restriction in ALE analysis. With these results, we also did not comment on either the neuroimaging predictors of improvement in depression with ECT or on the association of regions with significant change in rsFC with cognitive deficits developed after ECT. These aims need to be explored in separate meta-analyses.

## Conclusion and Future Directions

This meta-analysis aimed to understand the mechanisms of action of ECT in patients having depression. We focus on different measures of rsFC used in this group of patients and find those regions of cingulate gyrus showing a significant change with ECT, which has not changed often with other treatments for depression in earlier studies. These include reduction in rsFC in L. dACC and increase in rsFC of bilateral PCC. They are also noted in the literature as important predictors of improvement in depression with different treatments. In addition, we find increased rsFC in R. HC and R. PHG. Thus, our meta-analysis supports the argument of distinct mechanisms of action of ECT. The constraint in sample size and limitations in different aspects of neuroimaging of the studies included for this meta-analysis need to be addressed in future neuroimaging studies of ECT in depression. We also recommend the use of these regions to explore seed-based rsFC and to apply common measures of rsFC as rsfMRI studies in ECT are still in their early phase. Nonetheless, dynamic FC and GTN can be explored further on the rsfMRI data for studying the effect of ECT.

## Data Availability Statement

The original contributions presented in the study are included in the article/Supplementary Material, further inquiries can be directed to the corresponding author/s.

## Author Contributions

HJ did the ALE analysis and prepared figure and table related to ALE analysis. DI prepared the introduction. PS prepared other parts of manuscript including tables and figures, which was then reviewed by all of the authors. All three authors contributed to the systematic search and final selection of the articles and extraction of the data.

## Conflict of Interest

The authors declare that the research was conducted in the absence of any commercial or financial relationships that could be construed as a potential conflict of interest.

## Abbreviations

ACC: Anterior Cingulate Cortex
ALFF: Amplitude of Low-Frequency Fluctuation
ALE: Activation Likelihood Estimation
BA: Brodmann Area
BF: Bifrontal
BT: Bitemporal
CBMA: Coordinate based meta-analysis
CG: Cingluate Gyrus
DMN: Default Mode Network
dACC: dorsal Anterior Cingulate Cortex
dlPFC: dorsolateral Prefrontal Cortex
dmPFC: dorsomedial Prefrontal Cortex
ECT: Electroconvulsive therapy
fALFF: fractional Amplitude of Low-Frequency Fluctuation
FC: Functional Connectivity
FCD: Functional Connectivity Density
FcHo: Functional connectivity Homogeneity
FD: Framewise Displacement
FDR: False Discovery Rate
FCS: Functional Connectivity Strength
fMRI: functional magnetic resonance imaging
FWER: Family-wise Error Rate
FWHM: Full Width Half Maximum
gFCD: global Functional Connectivity Density
GMV: Gray matter volume
GTN: Graph Theory and Network analysis
HC: Hippocampus
IBMA: Image based meta-analysis
L.: Left
MNI: Montreal Neuroimaging Institute
PCC: Posterior Cingulate Cortex
PFC: Prefrontal Cortex
PHG: Parahippocampal Gyrus
R.: Right
ReHo: Regional Homogeneity
rsFC: resting state Functional Connectivity
rsfMRI: Resting state functional magnetic resonance imaging
ROI: Region of Interest with resting state functional connectivity
RUL: Right Unilateral
SDM: Signed differential mapping
SDM-PSI: Seed-based d mapping- permutation of subject imaging
sgACC: subgenual Anterior Cingulate Cortex
sbFC: Seed-based Functional Connectivity.
