# Supplementary material for: Resting State Functional Connectivity of Brain With Electroconvulsive Therapy in Depression: Meta-Analysis to Understand Its Mechanisms
Source: Front Hum Neurosci. 2021 Jan 21;14:616054. doi: 10.3389/fnhum.2020.616054 (PMC7859100; doi:10.3389/fnhum.2020.616054)
Supplement: Supplementary file 1 [file Table_1.DOCX]

**Supplementary Table 1:** Details of studies excluded from systematic review under ‘full-text’ phase

| **Author (Year)** | **Sample** | **Neuroimaging type; method and measure of analysis** | **Reason for Exclusion** |
| --- | --- | --- | --- |
| van Waarde et al. (2015) | Treatment resistant Unipolar depression, age > 18years, Brief pulses BT ECT | rsfMRI (BOLD); FC; Network based; ICA | Only baseline neuroimaging data available |
| Du et al. (2016) | Treatment naïve Unipolar depression, age > 18years, Brief pulse BT ECT | rsfMRI (BOLD); ALFF, fALFF; Voxel based | Single ECT session |
| Wolf et al. (2016) | ECT naïve depression or schizophrenia, age > 18 years, Brief pulse RUL ECT | structural MRI; Voxel based morphometry and surface based morphometry | fMRI was not done |
| Chen et al. (2017) | Treatment resistant depression, age > 18 years, Brief pulse BT ECT | rsfMRI (BOLD); FC; Network based; Group information guided ICA | No significant result was found |
| Thomann et al. (2017) | Treatment resistant depression or schizophrenia, age > 18 years, Brief pulse RUL ECT | structural MRI; voxel based morphometry  rsfMRI (BOLD); FC; voxel to voxel | Separate imaging data for Depression is not available |
| Leaver et al. (2018) | Treatment resistant Unipolar depression, age > 18 years , Brief pulse RUL/ RUL + BT ECT | rsfMRI (BOLD); ReHo, fALFF; Voxel based  rsfMRI (BOLD); GTN; ROI/ Network to ROI/ Network  rsfMRI (ASL); Cerebral blood flow; Voxel based | Only baseline neuroimaging data available |
| Moreno-Ortega et al. (2019) | Treatment resistant depression, age > 18 years, Ultra-brief pulse RUL | rsfMRI (BOLD); FC; Seed based within specific network  rsfMRI (BOLD); FC; Within network analysis | Only baseline neuroimaging data available |
| Oudega et al. (2019) | Late age onset (> 55 years) Unipolar depression, Brief pulse RUL/ RUL + BT | rsfMRI (BOLD); FC; Network based; ICA | Only baseline neuroimaging data available |
| Sambataro et al. (2019) | Treatment resistant depression or schizophrenia, age > 18 years, Brief pulse RUL ECT | rsfMRI (BOLD); FC; ROI/ Network based; ICA | Difference between pre and post ECT effects were not measured directly |
| Takamiya et al. (2019) | Any depression of age > 50 years, Brief pulse BT ECT | structural MRI; Grey matter volume  rsfMRI (BOLD); fALFF; Voxel based | Only baseline neuroimaging data available |

ALFF: Amplitude of Low-Frequency Fluctuation, ASL: Arterial Spin Labelling, BF: Bifrontal, BOLD: Blood Oxygen Level Dependent; BT: Bitemporal;

ECT: Electroconvulsive therapy, fALFF: fractional Amplitude of Low-Frequency Fluctuation, FC: Functional Connectivity, GTN: Graph Theory and Network analysis,

ICA: Independent Component Analysis, ReHo: Regional Homogeneity, rsfMRI: Resting state functional magnetic resonance imaging, ROI: Region of Interest,

RUL: Right Unilateral.

**Supplementary Table 2: Studies included in ALE analysis: Characteristics of Neuroimaging**

| **1^st^ Author (Year)**  **Scanning Characteristics**   - Manufacturer model, field strength, Eyes status - Pulse sequence, Number of volumes, Field of view, Matrix size, Slice thickness - Interslice skip, Acquisition orientation, TR, TE, Flip angle - Software (Name , Version) | **Preprocessing**   - Removal of initial volumes, Slice timing correction (STC), Distortion correction (DC) - Motion correction (MC) (Realignment with Number of translation parameters and function used) - Outlier Detection: (MSE/MSSD/ RMS), FD/ DVARS - Transformation model: Registration template details, Segmentation, Normalization, Reslicing, Smoothing (FWHM) - Denoising with linear regression: CSF-WM, Number of subject motion parameters, Identified outliers (scrubbing), Detrending (session effects), OR; TF | **Analysis**   - Specific strategy if used, Mask if used - Outcome analysis with Correction for Multiple comparisons | **Results Display**   - Tables/ Text   (coordinates, structure, Max-Z / t statistic, cluster size, specification of atlas   - Figure/ maps (Mask, regions- anatomy, thresholds) |
| --- | --- | --- | --- |
| **Abbott et al. (2013)**   - Siemens Trio, 3T, Eyes open with fixation - EPI (GE), 158 vol, 240*240mm^2^, 64*64, 3.5 mm - 1.05mm, **NA,** 2000ms, 29ms, 75° - SPM5, GIFT **(Version NA)** | - 4 vol, STC= Done, **DC= ND** - MC (**Realignment parameters details= NA**, Function used= INRIAlign (M-estimator) - **Outlier Detection: NA** - Normalized to MNI, Reslicing= 3 mm, FWHM=10 mm (for greater variability in older adult sample) - Denoising with Linear regression: CSF-WM and motion parameters by tCompCor, **OR=** ND, TF= 0.01 – 0.1 Hz | - RSN: Group ICA, Fisher transformation, Paired t-test for FNC changes between pre and post ECT; FDR correction, p< 0.05 - 2 samples t-test: Pre ECT Depression vs HC, Post ECT Depression vs HC - Correlation between ∆ECT FNC and HAM-D- Depression group**, uncorrected p < 0.05** - 2 factor ANOVA for longitudinal changes in FNC between group (ECT remitters and non-remitters) and time (pre and post ECT) | - Information: Present (coordinates, structure, Max-Z / t statistic, cluster size, specification of atlas) - Figures- Present (regions-anatomy presented with names, thresholds, t score bar, coordinate) |
| **Abbott et al. (2014)**   - Siemens Trio, 3T, Eyes open with fixation - EPI (GE), 154 vol, 240*240mm^2^, 64*64, 3.5 mm - 1.05mm**, NA,** 2000ms, 29ms, 75° - SPM8 | - **NA**, STC= Done, **DC= ND** - MC (**Realignment parameters details= NA**, Function used= INRIAlign (M-estimator) - **Outlier Detection: NA** - Normalized to MNI, Reslicing= 3 mm, FWHM=10 mm (for greater variability in older adult sample) - Denoising with Linear regression: CSF-WM and motion parameters by tCompCor, **OR=** ND, TF= 0.01 – 0.1 Hz | - Hippocampal Mask as ROI (intersection of the subject-specific mask and Wake Forest University Pick-Atlas hippocampal mask) - 2 samples t-test: Pre ECT Depression (responders) vs HC, Post ECT Depression (responders) vs HC - Paired-t test- Responders patients, pre and post ECT, **No covariates** - **FWER** (? Only for 2 samples t-test): **Not based on any model,** Cluster> 321 voxel, p < 0.05, cluster defining threshold > 0.005 - Correlation between ∆ECT FNC and HAM-D- whole patient group**, uncorrected p < 0.05** | - Information: Present (coordinates, structure, Max-Z / t statistic, cluster size, specification of atlas) - Figures- Present (regions-anatomy presented with names, thresholds, t score bar, coordinate) |
| **Liu et al. (2015)**   - GE Signa, 3T, Eyes closed - EPI (SE/GRE: NA), 200 volumes, 240*240 mm^2^, 64*64, 5 mm - **NA**, Axial (33), **NA**, 2000 ms, 30 ms, 90° - DPARSF **(Version NA),** REST (v1.8) | - 5 vol, STC= Done, **DC= ND,** - MC (Realignment= 3mm translation, 3 degree rotation)**,** **Function used= NA** - **Outlier Detection: NA** - fMRI to Anatomical to MNI Template, Reslicing 3 mm, FWHM= 6 mm - Denoising with Linear regression: CSF-WM, Global brain signal, 24 head motion parameters, **OR= ND**, TF= 0.01 – 0.08 Hz | - fALFF: Paired t test within gray matter mask; p < 0.05 (AlphaSim- corrected; height threshold p < 0.01, minimum cluster= 35) - seed (ALFF based) to voxel: paired t test within mask (regions in pre- and post-ECT by 1-sample t test within gray matter mask); p < 0.05 (AlphaSim- height threshold p < 0.01, minimum cluster= 24) - Pearson correlation: ∆ECT fALFF and HAM-D, ∆ECT ROI related Fz and HAM-D; **uncorrected p < 0.05** - ROI= 3 mm radius sphere centered at coordinates with peak T statistic - **No covariates added to any analysis** | - Tables: Present (coordinates, structure, Max-Z / t statistic, cluster size, specification of atlas) - Figures: Present (regions-anatomy presented with names, thresholds, t score bar); Absent (coordinate) |
| **Argyelan et al. (2016)**   - GE HDx, 3T, Eyes closed - **EPI (SE/ GE: NA),** 150 vol, 240*240mm^2^, 64*64, 3 mm - No Skip, Axial (40), 2000 ms, 30 ms, **NA** - 1000 Functional Connectomes Project based in FSL and AFNI | - 10 vol, **STC= ND, DC= ND** - MC Done (**Realignment details= NA)** - **Outlier Detection:** FD **(Threshold details NA)** - Registered and Normalized to MNI 152 (2mm) space, 12 parameter affine transformation, Reslicing= ND, FWHM=6 mm - Denoising with Linear regression: CSF-WM, 6 head motion parameters; **OR= ND;** Scrubbing, TF= 0.005 – 0.1 Hz | - fALFF and Seed to voxel: Repeated measures of ANOVA with subjects (independent across levels) and contrast over the main effect time. - fALFF**: FWER (Not based on any model, Minimum Cluster> 10, p < 0.05, cluster defining threshold- NA** - Seed to voxel FC: FDR corrected< 0.05, Minimum Cluster > 10 - Post-hoc analysis: Patients compared with controls across time points for both fALFF and seed to voxel FC, **uncorrected p < 0.05** - **fALFF: Also reported for uncorrected voxel level p<0.001** - **No covariates added to any analysis** | - Tables: Present (coordinates, structure, Max-Z / t statistic, cluster size, specification of atlas) - Figures- Present (regions-anatomy presented with names, thresholds, t score bar, coordinate) |
| **Leaver et al. (2016b)**   - Siemens Allegra, 3T, **NA** - **EPI** (SE/ GE: NA), 180 vol, 240*240mm^2^, **NA**, 5 mm - **NA**, Axial (34), 2000 ms, 30 ms, 70°   FSL v5.0, sSPM8, Brain Connectivity Toolbox **(Version NA)** | - **NA**, STC= Done**,** DC= Done by regflilt - MC (Realignment= 6 DF, middle vol) - **Outlier Detection= NA** - Registered and Normalized to MNI 152 (2mm) space, non-linear transformation, Reslicing= 2mm, FWHM=6 mm - Denoising with Linear regression: spin-history artifacts as regressor in FSL regflilt; Nuisance & Motion parameters not considered, **OR= ND**, High pass filter (0.01Hz), No low pass filter, | - ICA: 8 RSN defined - ∆ECT within single RSN: Linear mixed model for 8 RSN individually, subject= random factor, ∆ECT= fixed factor, 2 time points, FDR correction for voxel-wise threshold within single RSN, FWER for RSN (**random field cluster correction- details NA**), p_corr_ < 0.05 - ∆ECT in at least 3 RSN: Partial conjunction analysis, voxel-wise FDR, cluster size> 50 voxels, p_corr_ < 0.05 - ∆MDD: voxel-wise correlation analyses (Pearson's *r*) between change in rsFC & HAMD score; both rsFC for single and within 3 RSNs were analysed - GTN: Global network metrics calculated on ∆ECT & ∆Dep related significant ROI-ROI connections, 1sample t-test for each group separately (Depression & HC groups at pre & post ECT), p< 0.005 with Bonferroni correction for number of pairs | - Tables: Present (coordinates, structure, Max-Z / t statistic, cluster size, specification of atlas) - Figures: Present (regions-anatomy with names, thresholds); **Absent** (t score bar, coordinate) - **RSN: Regions and coordinates NA** |
| **Qiu et al. (2016)**   - GE Signa, 3T, Eyes closed - GRE- EPI, 205 volumes, 240*240 mm^2^, 64*64, 5 mm - **NA**, Axial (30), 2000 ms, 30 ms, 90° - DPARSF **(Version NA)** | - 10 vol, STC= Done, DC= **ND,** - MC (Realignment = 1.5 mm translation, 1.5° rotation, **Function used= NA**) - **Outlier Detection= NA** - Normalized directly to SPM8 echoplanar imaging template, Reslicing= 3 mm, FWHM= 4 mm - **Denoising with Linear Regression:? ND** - TF= 0.01 – 0.08 Hz | - **Analysis details at different level of GLM= NA** - **FWER corrected p value taken but details NA** | - Tables: Present (coordinates, structure, Max-Z / t statistic, cluster size, specification of atlas) - Figures: Present (t score bar, regions-anatomy presented but not named); **Absent** (thresholds, coordinate) |
| **Kong et al. (2017)**   - GE Signa HDx, 3T, Eyes closed - Std. EPI, Vol NA, 220*220 mm^2^, 64*64, 3.4 mm - No skip, Axial (33), 2000ms, 30ms, 90° - DPARSF **(Version NA),** REST **(Version NA)** | - 10 vol, STC= Done, **DC= ND,** - MC (Realignment= 2.5 mm translation, 2.5 degree rotation; **Function used= NA** - **Outlier Detection= NA** - fMRI to Anatomical to MNI template, Reslicing 3 mm, FWHM= 4 mm - Denoising with Linear regression: CSF-WM, 6 head motion parameters, Global brain signal, **OR= ND,** TF= 0.01 – 0.08 Hz | - ReHo: paired t-tests; Gaussian random field (GRF) method (voxel Z threshold < 0.005, cluster level p < 0.01, two- tailed) - ALFF: paired t-tests; GRF method (voxel Z threshold < 0.005, cluster level p < 0.01, two- tailed) - Pearson correlation; ∆ECT ReHo and HAM-D, ∆ECT ALFF and HAM-D; **uncorrected p < 0.05** - ROI= 6 mm radius sphere centered at coordinates with peak density region of paired t-test ReHo maps - **No covariates added to any analysis** | - Tables: Present (coordinates, structure, Max-Z / t statistic, cluster size, specification of atlas) - Figures: Present (thresholds, t score bar, z coordinate); **regions-anatomy presented but not named** |
| **Bai et al. (2018b)**  **AMHU**   - GE Signa HDx, 3T, Eyes closed - EPI (SE/ GE: NA), 240 Vol, 220*220mm^2^, 64*64, 3 mm - No Skip, Axial (33), **NA,** 2000 ms, 22.5 ms, 30°   **USTC**   - GE Discovery 750w, 3T, Eyes closed - EPI (SE/ GE: NA), 217 Vol, 192*192 mm^2^, 64*64, 3 mm - No Skip, Axial (46), **NA,** 2400 ms, 30 ms, 90° - DPARSF **(Version NA),** REST **(Version NA)**, SPM **(Version NA)** | - 10 vol, STC= Done, **DC= ND** - MC (Realignment= 1 voxel translation, 1 voxel rotation, **Function used= NA**) - Outlier Detection= FD > 0.5 - Coregistration, segmentation and normalized, **Template details= NA**, Reslicing= ND, FWHM= 4 mm - Denoising with Linear regression: Nuisance variables (CSF-WM only), 24 head motion parameters, FD + 1 time point prior to, and 2 time points following, each of these high motion time points, **OR= ND**, TF= 0.01 – 0.08 Hz | - For both ALFF and Seed to voxel FC analysis: 2-factor repeated-measure analysis of variance (ANOVA), group (depression and controls) repeated over time point (pre-ECT and post-ECT). Age, sex, number of ECT sessions, medicine load, and whole brain volume effects as covariates. Post hoc comparisons to detect group difference at TP1 (two-sample t test) and ECT effects for depressive patients and time effect for healthy controls (paired t test) - GRF method (voxel p < 0.001, cluster level p < 0.05, two- tailed); two-tailed p < .05 for interaction effect and p < .017 (0.05/3) for post hoc analyses - Exploratory Pearson correlation between ∆ECT brain function (ALFF, FC) and clinical response (HAM-D), **uncorrected p < 0.05** - ROI for seed to voxel: significant cluster in ALFF analysis | - Tables: Present (coordinates, structure, Max-Z / t statistic, cluster size, specification of atlas) - Figures- Present (regions-anatomy presented with names, thresholds, t score bar); **Absent (coordinate)** |
| **Qiu et al. (2019)**   - GE Signa HDx, 3T, Eyes closed - EPI (SE/ GE: NA), 200 Vol, 240*240mm^2^, 64*64, 5 mm - No Skip, Axial (33), **NA,** 2000 ms, 30 ms, 90°   DPABI **(version** **NA**), REST **(version** **NA**) | - 10 vol, STC= Done, DC= **ND,** - MC (Realignment = 1.5 mm (x, y, z), 1.5° rotation; **Function used= NA** - **Outlier Detection= NA** - Registered to the std. MNI space with normalization, Reslicing 3 mm, FWHM= 8 mm - **Denoising with Linear** **regression details= ? ND,** TF= 0.01 – 0.08 Hz, | - fALFF: paired t-test; AlphaSim- corrected (height threshold p < 0.01, minimum cluster= 85, cluster level threshold p < 0.05) | - Tables: Present (coordinates, structure, Max-Z / t statistic, cluster size, specification of atlas) - Figures- Present (regions-anatomy presented with names, t score bar); **Absent (thresholds, coordinate)** |
| **Wang et al. (2020b)**   - GE Signa HDx, 3T, Eyes closed - Std. EPI, 240 Vol, 220*220mm^2^, 64*64, 4 mm - No Skip, Axial (33), **NA,** 2000 ms, 22.5 ms, 30° - SPM8 | - 10 vol, STC= Done, **DC= ND** - MC (Realignment= 3mm translation, 3-degree rotation; **Function used= NA** - **Outlier Detection= NA** - fMRI to Anatomical to MNI Template, Reslicing= ND, FWHM= 6 mm - Denoising with Linear regression: CSF-WM, 6 head motion parameters, **OR= ND,** TF= 0.01 – 0.08 Hz | - Seed to Voxel FC: Insular sub regions used as seed= By tractographic Human Brainnetome Atlas - Paired 2 sample t- test (Depression and HC- pre and post ECT): cluster-level Monte Carlo simulation (5000 times) corrected threshold of p < .05 (cluster-forming threshold at voxel-level p < .001) - Granger causality analysis done to identify causal interaction between insula associated function- al networks (*not discussed here)* - Pearson correlation: ∆ECT regions of significant rsFC with insular subregion and HAMD, ∆ECT significant rsFC of among these regions and HAMD; **uncorrected p < 0.05** - **No covariates added to any analysis** | - Tables/text: Absent (coordinates, structure, Max-Z / t statistic, cluster size) - Figures- Present (regions-anatomy presented with names, thresholds), **Absent (t score bar, coordinate)** |
| **Takamiya et al. (2020)**   - GE Signa HDx, 3T, Eyes closed - EPI **(SE/GRE:NA),** 188 volumes, NA, 64*64, 3.5mm - **NA,** Axial (36), **NA,** 2000ms, 30ms, **NA** - CONN Functional Connectivity Toolbox **(Version NA)** with SPM 12 | - 10 vol, STC= Done, **DC= Done** - MC: Realignment details= **NA** - Outlier Detection= Artifact Rejection Toolbox (ART)- FD> 5mm - Normalized to MNI template; Reslicing= ND, FWHM= 8 mm - Denoising with Linear regression: CSF-WM by **aCompCor,** Global brain signal (z> 3), motion parameters (**number NA),** outliers, **OR= ND,** TF= 0.008–0.09 Hz | - Seed to voxel: Fisher-transformed Z scores of Subject-level seed maps used in 2nd level analysis; 1) A priori bilateral hippocampal seeds from Harvard-Oxford Atlas in CONN 2) MVPA based ROI - Contrasts for Seed to voxel and MVPA analysis: Pre-post ECT as between-conditions contrast, and percentage change in HAM-D as between-subjects contrast. Age, Sex and baseline HAM-D scores as covariates - Statistical threshold: cluster-level false discovery rate (FDR)-corrected p < 0.05 with a voxel height of p< 0.001 | - Tables: Present (coordinates, structure, Max-Z / t statistic, cluster size, specification of atlas) - Figures: Present (regions-anatomy presented with names); **Absent (thresholds, t score bar, coordinate)** |
| **Wang et al. (2020a)**   - GE, Model NA, 3T, Eyes closed - Std. EPI, 240 Vol, **NA,** 64*64, 4 mm - **NA,** Axial (33), **NA**, 2000 ms, 22.5 ms, 30° - Software used for analysis: **NA** | - 10 vol, STC= Done, **DC= ND** - MC (Realignment= 1voxel translation, 1voxel rotation), **Function used= NA** - Outlier Detection= FD >0.5 - Normalized to MNI template (Further details NA), Reslicing= ND, FWHM=6 mm - Denoising with Linear regression: CSF-WM, 24 head motion parameters, Outlier; **OR= ND**, TF= 0.01 – 0.08 Hz | - FcHo maps: paired t test; cluster-level Monte Carlo simulation (5000 times) corrected thresholds of p < .05 (cluster-forming threshold at voxel-level p < .001) - Seed (Significant changed FcHo area) to voxel: Fisher-transformed Z scores of Subject-level seed maps; 2 sample paired t test: cluster-level Monte Carlo simulation (5000 times) corrected threshold of P < .05 (cluster-forming threshold at voxel-level p < .001) - Pearson correlation; ∆ECT FcHo and HAM-D, ∆ECT rsFC and HAM-D ; p < 0.05. after Bonferroni correction - **No covariates added to any analysis** | - Tables: Present (coordinates, structure, Max-Z / t statistic, cluster size, specification of atlas) - Figures: Present (t score bar, regions-anatomy presented with names); **Absent (thresholds, coordinate); Only 1 Figure of result** |
| **Zhang 2020**   - GE Discovery 750w, 3T, Eyes closed - EPI (SE/ GE: NA), 217 Vol, 192*192 mm^2^, 64*64, 3 mm - No Skip, Axial (46), **NA,** 2400 ms, 30 ms, 90°s - DPARSF **(Version NA)** and REST **(Version NA)** on SPM 8 | - 5 vol, STC= Done, **DC= ND** - MC (Realignment Details= **NA)** - **Outlier Detection= NA** - Coregistration & Normalization= Anatomical to MNI Template (DARTEL) & then fMRI to Normalised Anatomical Figure, Reslicing= ND, FWHM= 4 mm - Denoising with Linear regression: CSF-WM, 24 head motion parameters, **OR= ND,** TF= 0.01 – 0.08 Hz, | - Seed to voxel: Two bilateral anterior insular subdivisions (ventral, dorsal) mapped to MNI 152; mean time series of all voxels in respective division as ROI; Pearson’s correlations of ROI with all other voxels in the whole brain - 2 sample t- test: HC and Depression groups; age, gender, education level and head motion as covariates within a gray matter mask; FWER: with voxel level p < 0.05 (two-tailed) and cluster threshold > 15. (**Not based on any model)** - **Paired sample t-tests**: Change in rsFC values (seed: vAI) pre-post ECT. **significant p< 0.05 without any correction** - Pearson correlation between ∆ECT rsFC & HAM-D, **significant p < 0.05 without any correction** | - Tables/text: Present (coordinates, structure, Max-Z / t statistic, cluster size) - Figures- Present (regions-anatomy presented with names, threshold, t score bar, coordinate) |

**ALFF:** Amplitude of Low-Frequency Fluctuation, **CSF-WM:** Cerebrospinal fluid- White matter, **DC:** Distortion correction, **DARTEL:** Diffeomorphic Anatomical Registration Through Exponentiated Lie algebra , **DPABI:** Data Processing & Analysis for (resting state) Brain Imaging, **DPARSF:** Data Processing Assistant for rs-fMRI, **ECT:** Electroconvulsive therapy, **EPI (GE):** Echoplanar imaging (Gradient echo), **fALFF:** fractional Amplitude of Low-Frequency Fluctuation**, FC:** Functional Connectivity, **FCD:** Functional Connectivity Density**, FcHo:** Functional connectivity Homogeneity, **FD**: Framewise displacement, **FDR:** False discovery rate, **FEWR:** Family wise Error rate, **FNC:** Functional network connectivity, **FSL:** Functional, structural and diffusion brain image (FMRIB) Software Library, **FWHM:** Full width at Half oof Maximum, **GIFT:** Group ICA fMRI Toolbox, **GRF:** Gaussian random field, **, GTN:** Graph Theory and Network analysis, **HAM-D:** Hamilton depression rating scale, **HC:** Healthy control, **ICA:** Independent Component Analysis, **MC:** Motion correction, **MNI:** Montreal Neuroimaging Institute, **MSE/ MSSD/ RMS:** Mean square error/ Mean square successive difference/ Root mean square (for temporal variance in image intensity), **MVPA:** Multivoxel pattern analysis, **NA:** Information not available, **ND:** Not Done, **OR:** Orthogonalization of regression, **ReHo:** Regional Homogeneity, **ROI:** Region of Interest, **rsFC:** resting state Functional Connectivity, **REST:** Resting State fMRI Data Analysis Toolkit, **RSN:** Resting state network, **SPM:** Statistical Parametric Mapping, **STC:** Slice timing correction, **TE:** Echo time i.e. Time between middle of exciting RadioFrequency pulse and middle of spin echo production, **TF:** Temporal Filtering, **TR:** Repetition time for the whole pulse sequence in MRI, **vAI:** ventral Anterior Insula

**Supplementary Table 3:** Studies included in ALE meta-analysis: Detailed results of neuroimaging analysis of rsFC for pre and post ECT scans

| **1st Author (Year)** | **rsFC measure, extraction method, seed (if ussed)** | **Coordinates/ Area/ number of voxels** | **t score/ p value, Direction of significance** |
| --- | --- | --- | --- |
| **Abbott et al. (2013)** | - FC, RSN to RSN | - Na/ posterior DMN- dorsomedial PFC/ Na - Na/ posterior DMN- L. dorsolateral PFC/ Na | - 5.38, Post> pre - 3.85, Post> pre   Both changed from -ve to +ve correlation |
| **Abbott et al. (2014)** | - FC, Seed to Voxel, R HC | - 42, -22, -11/ R. TL/ 307 | - p_FWER_= 0.025, Post> pre |
| **Liu et al. (2015)** | - ALFF, Voxel to Voxel | - -30, -6, -18/ L. HC/ 38 - -9, 24, -15/ L. subgenual ACC/ 137 - -21, 57, -15/ L. mid OFC/ 43 - 48, 39, -15/ R. inf OFC/ 221 - 15, 42, 3/ R. pregenual ACC/ 42 - 45, -12, 48/ R. Precentral Gyrus/ 161 - -3, -9, 45/ L. post dorsal ACC/ 43 | - 4.32/ post> pre - 4.6/ post> pre - 5.28/ post> pre - 4.88/ post> pre - 5.19/ post> pre - 5.22/ post> pre - -4.54/ pre> post |
|  | - FC, Seed to voxel, L. sgACC | - 45, 9, -36/ R. MTP/ 105 - 33, 51, -15/ R. mid OFC/ 24 - 36, 30, -9/ R. inf OFC/ 50 - -12, 30, -21/ L. sup OFC/ 48 - -21, 6, -24/ L. PHG/ 25 - -9, 39, 3/ L. pregenual ACC/ 84 | - 5.36/ post> pre - 4.87/ post> pre - 3.68/ post> pre - 4.54/ post> pre - 4.11/ post> pre - 3.92/ post> pre |
| **Argyelan et al. (2016)** | - fALFF, Voxel to Voxel^1^ | - 8, 26, − 6, R. SCC, 32 | - -6.81, pre> post |
|  | - FC, Seed to Voxel, R. SCC | - -2, 52, -10, ventromedial PFC, 415 - -34, -32, -26, L. PHG, 428 - 30, -28, -24, R. PHG, 168 - -60, 8, -6, L. Temporal Pole, 387 - 44, 10, -18, R. Temporal Pole, 563 - 46, -44, 14, R. SMG, 129 | - -4.93, pre> post - -5.03, pre> post - -5.28 pre> post - -5.82, pre> post - -4.93, pre> post - -4.85, pre> post |
| **Leaver et al. (2016b)** | - FC, RSN to ROI (single RSN) | - -5.3, 12.3, 30.0; Th/ ventral Basal Ganglia Network- dorsal ACC; 266 - 6.2, -58.1, 22.7; ant DMN- PCC; 104 - -2.0, -18.1, 7.0; SAL- mdTh; 93 - 17.1, -55.4, -19.2; SAL- R. lat CBL; 69 - 21.4, -30.4, -10.8; posterior DMN- R. HC; 61 | - NA, post> pre - NA, post> pre - NA, post> pre - NA, post> pre - NA, post> pre (from -ve to +ve correlation) |
|  | - FC, RSN to ROI (At least 3RSN) | - -0.5, 11.8, 26.5; dorsal ACC; 122 - -2.5, -41.3, 9.6; PCC; 93 - 38.4, -8.7, 43.7; R. ATL; 69 - 2.5, -54.5, -33.6; Precuneus; 65 - -4.8, -17.5, 6.9; mdTh; 65 - 12.8, -61.2, 47.6; medPC; 51 | Na (These ROIs were connected to multiple RSN) |
| **Qiu et al. (2016)** | - ReHo, Voxel to Voxel | - 42, 42, –3, (R. mid & inf FG), 41 - 6, 9, 9, R. Caudate, 37 - -36, -78, 45, (L. AG, L. Precuneus, L. medial FG), 53 - 57, -57, 36, (R. SMG, R. AG), 46 - -18, 27, 48, (L. sup FG, L. mid FG), 33 - -33, -57, -33, L. ant CBL, 40 - 57, -3, -9, (R. mid & sup TG), 38 - 12, 9, 42, R. CG, 21 | - 4.68, post> pre - 4.03, post> pre - 4.43, post> pre - 4.10, post> pre - 5.14, post> pre - -3.92, pre> post - -5.29, pre> post - -5.06, pre> post |
| **Kong et al. (2017)** | - ReHo, Voxel to Voxel | - -12, 54, 39, L. sup FG, 91 - 24, 39, 54, R. sup FG, 90 | - -6.54, pre> post - -7.77, pre> post |
|  | - ALFF, Voxel to Voxel | - -36, 63, 6, L. mid FG, 323 - 21, 42, -21, R. mid FG (orbital part), 94 - -6, 21, 36, L. mid CG, 133 - -30, 0, 45, L. Precentral Gyrus, 118 - 21, 6, 48, R sup & mid FG, 107 | - 7.93, post> pre - 6.10, post> pre - -6.74, pre> post - -5.71, pre> post - -6.21, pre> post |
| **Bai et al. (2018b)**  **(AMHU)** | - ALFF, Voxel to Voxel | - 63, -42, -27, R. inf TG, 36 - 3, 36, 51, R. dmPFC, 22 - -12, -54, 72, L. Postcentral Gyrus extending to Precuneus, 331 - -42, -57, -24, L. CBL, 25 - 39, 3, -6, R. sup TG/ insula, 36 - 48, -45, 0, R. mid/sup TG, 22 - -3, -45, 45, L. Precuneus/ PCC, 26 - -12, -54, 63, L. Precuneus/ PCC, 71 - -21, -69, 6, L. Calcarine, 20 - -9, 24, 30, L. dorsal ACC, 21 | - 6.86, post> pre - 5.63, post> pre - 6.78, post> pre - -4.74, pre> post - -5.54, pre> post - -4.68, pre> post - -5.39, pre> post - -6.20, pre> post - -4.50, pre> post - -5.92, pre> post |
|  | - FC, Seed to Voxel, dmPFC | - -60, -36, -27, L. inf/mid TG, 79 - 6, 69, -3, R. OFC, 42 - 27, 66, -6, L.OFC, 35 - -33, -69, 27, L. TPJ extending to OG, 291 - -6, -45, 27, PCC, 101 - 39, -60, 24, R. TPJ - 3, 54, 36, R. dorsomedial PFC | - 5.64, post> pre - 4.72, post> pre - 5.09, post> pre - 7.12, post> pre - 5.43, post> pre - 4.74, post> pre - 5.53, post> pre |
| **Bai et al. (2018b)**  **(USTC)** | - ALFF, Voxel to Voxel | - 0, 57, 36, R. dorsomedial PFC, 36 | - 7.05, post> pre |
|  | - FC, Seed to Voxel, dmPFC | - 6, -45, 39, PCC, 127 - 39, -63, 42, R. inf TG, 100 | - 6.80, post> pre - 5.53, post> pre |
| **Qiu et al. (2019)** | - fALFF, Voxel to Voxel | - 9, -36, -33; R. CBL; 89 - -30, -24, -9; L. Insula, PHG; 186 - 39, 36, -6; R inf & mid FG; 85 | - -3.74; pre>post - -3.9; pre>post - -3.98; pre>post |
| **Wang et al. (2020b)** | - FC, Seed to Voxel, R. ant Insula (ventral) | - 18, 21, 45, B/L dorsolateral PFC, 716 - 42, -54, 33, R. AG, 143 - -45, -69, 36, L. AG, 139 - 21, -3, 21, B/L Caudate, 142 | - 6.98, post> pre - 5.81, post> pre - 5.14, post> pre - 5.85, post> pre |
| **Takamiya et al. (2020)** | - MVPA, Voxel to Voxel | - 28, -4, -20, R. HC/Amyg, 140 - -10, -54, 68, L. Precun, 123 - 18, -28, 8, R. Th, 107 - 46, -68, 34, R. AG, 100 - -24, -82, 42, L. OG, 89 - 54, -8, 38, R. Precentral gyrus/ Postcentral Gyrus, 86 | - NA - NA - NA - NA - NA - NA |
|  | - FC, Seed to Voxel, R HC - FC, Seed to Voxel, R HC/ Amygdala | - 0, 34, -20, (B/L SCC, B/L medial FG, R. OFC), 500 - 32, -2, 58, R. sup FG, 259 - −12, 32, 62, L. sup FG, 225 - −40, 14, 50, L. mid FG, 252 - 2, 50, −22, B/L SCC, 231 - −40, −66, 38, L. AG, 664 - 48, 24, 44, R. mid FG, 135 - −2, −46, 36, B/L Precuneus, 162 - −56,−32, 12, L. Planum Temporale & Parietal Operculum, 386 | - 6.60, pre> post - 5.52, post> pre - -6.90, pre> post - -6.81, pre> post - -6.35, pre> post - -5.58, pre> post - -4.78, pre> post - -4.45, pre> post - 6.29, post> pre |
| **Wang et al. (2020a)** | - FcHo, Voxel to Voxel | - 9, 42, 39, dmPFC, 76 - -45, -54, 36, L. AG, 86 | - 5.64, post> pre - 5.09, post> pre |
|  | - Fc, Seed to Voxel, dmPFC - Fc, Seed to Voxel, L. AG | - 18, 51, 18, R. sup FG, 348 - 48, -48, 27, R. AG, 347 - 6, -63, 48, R. Precuneus, 305, - 42, 15, 24, R. dorsolateral lPFC, 165 - -45, 36, 6, L. ventrolateral PFC, 230 - 48, 21, 30, R. dorsolateral PFC, 246 - -30, 12, 60, L. dorsolateral PFC, 552 - 45, -69, 42, R. AG, 365 | - 6.81, post> pre - 5.41, post> pre - 5.02, post> pre - 5.25, post> pre - 6.4, post> pre - 5.29, post> pre - 7.1, post> pre - 5.9, post> pre |
| **Zhang et al. (2020)** | - FC, Seed to Voxel, R. anterior Insula (ventral) - FC, Seed to Voxel, L. anterior Insula (ventral) | - 21, 24, -6, R. OFC, 19 - 21, 18, -12, R. OFC, 52 | - 3.41, post> pre - 2.95, post> pre |

**Note:** Results are described here only for patient group, with respect to rsFC as pre to post ECT change.

Results NOT described here are- (i) Healthy control group: All analysis (ii) Patients group: Related to correlation of RsFC to clinical improvement or cognitive functions change, and Other neuroimaging findings besides RsFC

**ACC:** Anterior Cingulate Cortex, **AG:** Angular Gyrus, **ALFF:** Amplitude of Low-Frequency Fluctuation, **AMHU:** Anhui Mental Health Center as Study site, **ant:** Anterior, **ATL:** Anterior Temporal Lobe, **B/L:** Bilateral, **CBL:** Cerebellum, **CG:** Cingulate Gyrus, **DMN:** Default Mode Network, **fALFF:** fractional Amplitude of Low-Frequency Fluctuation**, FC:** Functional Connectivity, **FCD:** Functional Connectivity Density**, FcHo:** Functional connectivity Homogeneity, **FNC:** Functional network connectivity, **FG:** Frontal Gyrus, **GTN:** Graph Theory and Network analysis, **HC:** Hippocampus, **inf:** Inferior, **L.**: Left, **lat:** Lateral, **mdTh:** mediodorsal Thalamus, **mid:** Middle, **MVPA:** Multivoxel pattern analysis, **NA:** Information not available, **Na:** Not Applicable, **OFC:** Orbitofrontal cortex, **OG:** Occipital Gyrus, **PCC:** Posterior Cingulate Cortex, **PFC:** Prefrontal Cortex, **PHG:** Parahippocampal Gyrus, **pre> post:** Greater before the ECT course, **post> pre:** Greater after the ECT course/ specified number of ECT sessions**, post:** Posterior, **R.:** Right, **ReHo:** Regional Homogeneity, **ROI:** Region of Interest, **rsFC:** resting state Functional Connectivity, **RSN:** Resting state network, **SAL:** Salience Network, **SCC:** Subcallosal Cingulate Cortex, **SMG:** Supramarginal Gyrus, **sup:** Superior, **Th:** Thalamus, **TG:** Temporal Gyrus, **TPJ:** Temporo-parietal Junction, **USTC:** University of Science and Technology of China as Study site.

**Supplementary Table 4**: List of studies excluded from ALE analysis: Clinical characteristics and rsFC findings

| **1^st^ Author (Year)** | **Clinical & Treatment Characteristic**   - **Total No., Age as Mean (SD), M:F, Disease details** - **Pulse Width, Electrode Placement, Frequency of ECT session, Session with Post ECT MRI- (Fixed no/ Last session), Anesthetic, Muscle relaxant** | **rsFC measure, extraction method, seed (if used)** | **Findings**  **(Regions; direction of finding)** |
| --- | --- | --- | --- |
| **Beall et al. (2012)** | - 6, 39.0 (5.4), 4:2, Treatment resistant depression - **NA,** BT, 3 times a week, Last session- 8.8(3.9), **NA, NA** | rsFC, ROI based (9 cortical and 2 subcortical B/L ROI)**^1^** | - L. ACC- OFC; pre> post - R. ACC- OFC; post> pre - ACC- Caudate; post> pre |
| **Perrin et al. (2012)** | - 9, 46.8 **(NA),** 6:3, Treatment resistant depression - Brief, BT, 2 times a week, Last session- 8.3 **(NA),** Propofol, S. Ch | weighted FC, Voxel based | - L. dlPFC, pre> post |
| **Wei et al. (2014)** | - 11, 35.45 (10.18), 7:4, UPD - Brief, BF, 1st 3 daily, then 3 times a week, Last session- 6.82 (2.80), Propofol, S. Ch | rsFC, Voxel based as VMHC | - B/L supFG, B/L midFG, B/L AG; post> pre |
| **Cano et al. (2016)^2^** | - 15, 57.73 (11.85), 7:8, Treatment resistant UPD - **NA,** BT**, NA,** 9^th^ Session, Thiopentone, S. Ch | rsFC, Seed to ROI (R. cm/sfAmyg) within B/L sgACC and dlPFC masks | - L. sgACC; pre> post |
| **Leaver et al. (2016a)^3^** | - 24, NA, NA, Treatment resistant depression - NA, RUL/ RUL+BF, 3 times a week, Last session- **NA** | Corticolimbic RSN to voxel | - vDMN (PCC)- VS; pre> post - aDMN (mPFC)- VS; post> pre^4^ |
| **Mulders et al. (2016)^5^** | - 16, 49.6(9.29), 6:10, Treatment resistant UPD - Brief, BT, 2 times a week, Last session- 17.8 (6.8), Etomidate, S. Ch. | rsFC as Variance ratio within DMN mask, Voxel to Voxel | - R. lateral TG |
| **Wang et al. (2017)** | - 23, 38.74 (11.02), 11:12, UPD (Treatment resistance or for suicide) - **NA**, BF, 1^st^ 3 daily, then 3 times a week; Last session- 7.36 (2), Propofol, S Ch | rsFC, Seed based (L. sfAmyg) | - L. post FFA; post> pre |
| **Bai et al. (2018a)** | - 45, 38.02 (11.65), 17: 28, Any depression - Brief, BT, 3 times a week, Last session- 5.16 (4.49), Propofol, S. Ch | rsFC, Seed based, L. antHT | - L. midOG, L. Putamen, R. medTG; post> pre |
| **Wang et al. (2018a)** | - 23, 38.74 (11.02), 11:12, UPD (Treatment resistance or for suicide) - **NA**, BF, 1^st^ 3 daily, then 3 times a week; Last session- 7.36 (2), Propofol, S Ch | - rsFC, RSN based (ICA) - rsFC, ROI to ROI | - Intranetwork: ECN; - Internetwork: DMN-SAL, DMN-ECN, SAL-DAN; - PCC- R. IPS, L. posterior CBL, R. anterior PFC - R. IPS- mPFC, L. ACC - L. LP- dmPFC, L. anterior PFC, R. ACC - dmPFC- L. anterior PFC   All had post> pre |
| **Wang et al. (2018b)** | - 23, 38.74 (11.02), 11:12, UPD (Treatment resistance or for suicide) - **NA**, BF, 1^st^ 3 daily, then 3 times a week; Last session- 7.36 (2), Propofol, S Ch | - Local FCD, Voxel based - rsFC, Seed based, R. supTG | - B/L precentral gyrus/ postcentral gyrus, B/L supTG; post> pre - R. IPS |
| **Wei et al. (2018)** | - 26, **NA, NA,** UPD - Brief, Bifrontal, 1st 3 daily, then 3 times a week, Last session- NA, Propofol, S. Ch | - rsFCS, Voxel based | - L. AG; post> pre |
| **Li et al. (2019)** | - 24, 32.5 (11.7), 10:14, UPD - Brief, BT, 1^st^ 3 daily, then 3 times a week, 8^th^ session, Propofol, S. Ch | global FCD, Voxel based | - R. Insula, pre> post |
| **Sinha et al. (2019)** | - 17, 44.8(18.9), 7:10, Any depression - Brief, BF, 3 times a week, 6^th^ session, Thiopentone, S.Ch. | GTN (Clustering Coefficient) | - L. inf.FO, L. medial supFG, L. Paracentral lobule, R. Pallidum; post> pre |
| **Leaver et al. (2020)** | - 17, 43.53 (13.17), 7:10, Treatment resistant depression (Responders) - Ultra-brief, RUL (15)/ RUL+BT(2), 3 times a week, Last session- 10.35 (2.62) | GTN of 3 RSN of HC (CBF based 3 seeds to get its FC) | - Middle HC-Th-BG network: Network Strength; pre> post |
| **Qi et al. (2020)** | - 118 (UNM: 75, UCLA: 43), 56.2 (16.0), 43:71, UNM: UPD only, UCLA: UPD= 36, BPD= 7 - Brief, RUL (81)/ RUL+BT (37), **NA**, Last session- 11 (3.4) | - sMRI (GMV) fused with rsfMRI (fALFF) through “MCCAR + jICA” | - PFC, insula, caudate, HC; pre> post |
| **Sun et al. (2020)** | - 122, 56.3(15.9), 43:79, Any depression - Brief, RUL/ RUL+BT, 3 times a week, Last session- **NA,** Methohexital/ Etomidate, S. Ch. | rsFC, ROI based | infFG- supFG, Cingulate gyrus- midFG, IPL- OrG, and latOG / midFG; post> pre |
| **Wei et al. (2020b)** | - 28, 37.12 (11.53), 12:16, UPD - Brief, BF, 1st 3 daily, then 3 times a week, Last session- 7.64 (1.95), Propofol, S. Ch | rsFC with cerebellum mask, seed based, L. sgACC | - L. CBL lobule VI; post> pre |
| **Wei et al. (2020a)** | - 28, 37.12 (11.53), 12:16, UPD - Brief, BF, 1st 3 daily, then 3 times a week, Last session- 7.64 (1.95), Propofol, S. Ch | - rsFC, Seed based (within Th), Parietal Cortex - rsFC, Seed based, L. Pulvinar | - L. Pulvinar; post> pre - B/L Precuneus; post> pre |

**Note:** Results are described here only for patient group, with respect to rsFC as pre to post ECT change.

Results NOT described here are- (i) Healthy control group: All analysis (ii) Patients group: Related to correlation of rsFC to clinical improvement or cognitive functions change, and Other neuroimaging findings besides rsFC

**AG:** Angular gyrus, **aDMN:** anterior DMN, **BF**: Bifrontal, **B/L:** Bilateral, **BPD**: Bipolar depression, **BT**: Bitemporal, **CBF**: Cerebral Blood Flow, **CBL:** Cerebellum**, ECN:** Executive control network, **fALFF:** fractional Amplitude of Low-Frequency Fluctuation, **FCD:** Functional Connectivity Density, **GMV:** Grey matter volume, **GTN:** Graph Theory and Network analysis, **HAM-D:** Hamilton Depressing rating Scale, **ICA:** Independent component analysis, **infFG:** inferior Frontal gyrus, **infFO:** inferior Frontal operculum, **IPL:** inferior Parietal lobule, **IPS:** Intra parietal sulcus, **latOG:** lateral Occipital gyrus**, midFG:** middle Frontal gyrus, **NA:** Information not available, **MCCAR + jICA**: multisite canonical correlation analysis with reference + joint independent component analysis, **OFC:** Orbitofrontal cortex, **OrG:** Orbital gyrus, **pre> post**: Greater before the ECT course, **post> pre**: Greater after the ECT course/ specified number of ECT sessions, **rsFC:** Resting state Functional Connectivity, **ROI:** Region of Interest with resting state functional connectivity, **rsFCS:** Resting state Functional Connectivity Strength, **RUL:** Right Unilateral, **SAL:** Salience network**,** **SD:** Standard deviation, **sMRI:** structural MRI, **supFG:** superior Frontal gyrus, **supTG**: superior Temporal gyrus, **RSN:** Resting state network, **S. Ch:** Succinylcholine, **UCLA:** Study site as University of California Los Angeles, **UNM:** Study site as University of New Mexico, UPD: Unipolar Depression, **VHMC:** Voxel-Mirrored Homotopic Connectivity, **vDMN**: ventral DMN

^1^Not mentioned clearly whether Bonferroni correction for multiple comparison was done or not for rsFC analysis

^2^15 patients came for MRI before and after 1^st^ ECT session, but only 13 patients came for MRI after 9^th^ ECT session; L. cm/sfAmyg did not have any significant change in connectivity with either sgACC or dlPFC.

^3^33 patients came for MRI before 1^st^ ECT and after 2nd ECT sessions, but only 24 patients came for MRI after their last ECT session

^4^Significant after correction for multiple comparisons was done only for post 2^nd^ ECT session but not for post last ECT session

^5^Only FDR corrected and cluster threshold considered= 20 voxels size

**REFERENCES**

Abbott, C.C., Jones, T., Lemke, N.T., Gallegos, P., McClintock, S.M., Mayer, A.R., et al. (2014). Hippocampal structural and functional changes associated with electroconvulsive therapy response. *Transl Psychiatry* 4**,** e483. doi: 10.1038/tp.2014.124.

Abbott, C.C., Lemke, N.T., Gopal, S., Thoma, R.J., Bustillo, J., Calhoun, V.D., et al. (2013). Electroconvulsive therapy response in major depressive disorder: a pilot functional network connectivity resting state FMRI investigation. *Front Psychiatry* 4**,** 10. doi: 10.3389/fpsyt.2013.00010.

Argyelan, M., Lencz, T., Kaliora, S., Sarpal, D.K., Weissman, N., Kingsley, P.B., et al. (2016). Subgenual cingulate cortical activity predicts the efficacy of electroconvulsive therapy. *Transl Psychiatry* 6**,** e789. doi: 10.1038/tp.2016.54.

Bai, T., Wei, Q., Xie, W., Wang, A., Wang, J., Ji, G.J., et al. (2018a). Hippocampal-subregion functional alterations associated with antidepressant effects and cognitive impairments of electroconvulsive therapy. *Psychol Med***,** 1-8. doi: 10.1017/S0033291718002684.

Bai, T., Wei, Q., Zu, M., Xie, W., Wang, J., Gong-Jun, J., et al. (2018b). Functional plasticity of the dorsomedial prefrontal cortex in depression reorganized by electroconvulsive therapy: Validation in two independent samples. *Hum Brain Mapp*. doi: 10.1002/hbm.24387.

Beall, E.B., Malone, D.A., Dale, R.M., Muzina, D.J., Koenig, K.A., Bhattacharrya, P.K., et al. (2012). Effects of electroconvulsive therapy on brain functional activation and connectivity in depression. *The Journal of ECT* 28(4)**,** 234-241. doi: 10.1097/YCT.0b013e31825ebcc7.

Cano, M., Cardoner, N., Urretavizcaya, M., Martinez-Zalacain, I., Goldberg, X., Via, E., et al. (2016). Modulation of Limbic and Prefrontal Connectivity by Electroconvulsive Therapy in Treatment-resistant Depression: A Preliminary Study. *Brain Stimul* 9(1)**,** 65-71. doi: 10.1016/j.brs.2015.08.016.

Chen, G.-D., Ji, F., Li, G.-Y., Lyu, B.-X., Hu, W., and Zhuo, C.-J. (2017). Antidepressant Effects of Electroconvulsive Therapy Unrelated to the Brain's Functional Network Connectivity alterations at an Individual Level. *Chinese medical journal* 130(4)**,** 414-419. doi: 10.4103/0366-6999.199845.

Du, L., Qiu, H., Liu, H., Zhao, W., Tang, Y., Fu, Y., et al. (2016). Changes in Problem-Solving Capacity and Association With Spontaneous Brain Activity After a Single Electroconvulsive Treatment in Major Depressive Disorder. *J ECT* 32(1)**,** 49-54. doi: 10.1097/YCT.0000000000000269.

Kong, X.M., Xu, S.X., Sun, Y., Wang, K.Y., Wang, C., Zhang, J., et al. (2017). Electroconvulsive therapy changes the regional resting state function measured by regional homogeneity (ReHo) and amplitude of low frequency fluctuations (ALFF) in elderly major depressive disorder patients: An exploratory study. *Psychiatry Res* 264**,** 13-21. doi: 10.1016/j.pscychresns.2017.04.001.

Leaver, A.M., Espinoza, R., Joshi, S.H., Vasavada, M., Njau, S., Woods, R.P., et al. (2016a). Desynchronization and Plasticity of Striato-frontal Connectivity in Major Depressive Disorder. *Cereb Cortex* 26(11)**,** 4337-4346. doi: 10.1093/cercor/bhv207.

Leaver, A.M., Espinoza, R., Pirnia, T., Joshi, S.H., Woods, R.P., and Narr, K.L. (2016b). Modulation of intrinsic brain activity by electroconvulsive therapy in major depression. *Biol Psychiatry Cogn Neurosci Neuroimaging* 1(1)**,** 77-86. doi: 10.1016/j.bpsc.2015.09.001.

Leaver, A.M., Vasavada, M., Kubicki, A., Wade, B., Loureiro, J., Hellemann, G., et al. (2020). Hippocampal subregions and networks linked with antidepressant response to electroconvulsive therapy. *Mol Psychiatry*. doi: 10.1038/s41380-020-0666-z.

Leaver, A.M., Wade, B., Vasavada, M., Hellemann, G., Joshi, S.H., Espinoza, R., et al. (2018). Fronto-Temporal Connectivity Predicts ECT Outcome in Major Depression. *Front Psychiatry* 9**,** 92. doi: 10.3389/fpsyt.2018.00092.

Li, X., Meng, H., Fu, Y., Du, L., Qiu, H., Qiu, T., et al. (2019). The Impact of Whole Brain Global Functional Connectivity Density Following MECT in Major Depression: A Follow-Up Study. *Front Psychiatry* 10**,** 7. doi: 10.3389/fpsyt.2019.00007.

Liu, Y., Du, L., Li, Y., Liu, H., Zhao, W., Liu, D., et al. (2015). Antidepressant Effects of Electroconvulsive Therapy Correlate With Subgenual Anterior Cingulate Activity and Connectivity in Depression. *Medicine (Baltimore)* 94(45)**,** e2033. doi: 10.1097/MD.0000000000002033.

Moreno-Ortega, M., Prudic, J., Rowny, S., Patel, G.H., Kangarlu, A., Lee, S., et al. (2019). Resting state functional connectivity predictors of treatment response to electroconvulsive therapy in depression. *Sci Rep* 9(1)**,** 5071. doi: 10.1038/s41598-019-41175-4.

Mulders, P.C., van Eijndhoven, P.F., Pluijmen, J., Schene, A.H., Tendolkar, I., and Beckmann, C.F. (2016). Default mode network coherence in treatment-resistant major depressive disorder during electroconvulsive therapy. *J Affect Disord* 205**,** 130-137. doi: 10.1016/j.jad.2016.06.059.

Oudega, M.L., van der Werf, Y.D., Dols, A., Wattjes, M.P., Barkhof, F., Bouckaert, F., et al. (2019). Exploring resting state connectivity in patients with psychotic depression. *PLoS One* 14(1)**,** e0209908. doi: 10.1371/journal.pone.0209908.

Perrin, J.S., Merz, S., Bennett, D.M., Currie, J., Steele, D.J., Reid, I.C., et al. (2012). Electroconvulsive therapy reduces frontal cortical connectivity in severe depressive disorder. *Proc Natl Acad Sci U S A* 109(14)**,** 5464-5468. doi: 10.1073/pnas.1117206109.

Qi, S., Abbott, C.C., Narr, K.L., Jiang, R., Upston, J., McClintock, S.M., et al. (2020). Electroconvulsive therapy treatment responsive multimodal brain networks. *Hum Brain Mapp* 41(7)**,** 1775-1785. doi: 10.1002/hbm.24910.

Qiu, H., Li, X., Luo, Q., Li, Y., Zhou, X., Cao, H., et al. (2019). Alterations in patients with major depressive disorder before and after electroconvulsive therapy measured by fractional amplitude of low-frequency fluctuations (fALFF). *J Affect Disord* 244**,** 92-99. doi: 10.1016/j.jad.2018.10.099.

Qiu, H., Li, X., Zhao, W., Du, L., Huang, P., Fu, Y., et al. (2016). Electroconvulsive Therapy-Induced Brain Structural and Functional Changes in Major Depressive Disorders: A Longitudinal Study. *Med Sci Monit* 22**,** 4577-4586. doi: 10.12659/msm.898081.

Sambataro, F., Thomann, P.A., Nolte, H.M., Hasenkamp, J.H., Hirjak, D., Kubera, K.M., et al. (2019). Transdiagnostic modulation of brain networks by electroconvulsive therapy in schizophrenia and major depression. *Eur Neuropsychopharmacol* 29(8)**,** 925-935. doi: 10.1016/j.euroneuro.2019.06.002.

Sinha, P., Reddy, R.V., Srivastava, P., Mehta, U.M., and Bharath, R.D. (2019). Network neurobiology of electroconvulsive therapy in patients with depression. *Psychiatry Research: Neuroimaging* 287**,** 31-40. doi: <https://doi.org/10.1016/j.pscychresns.2019.03.008>.

Sun, H., Jiang, R., Qi, S., Narr, K.L., Wade, B.S., Upston, J., et al. (2020). Preliminary prediction of individual response to electroconvulsive therapy using whole-brain functional magnetic resonance imaging data. *Neuroimage Clin* 26**,** 102080. doi: 10.1016/j.nicl.2019.102080.

Takamiya, A., Kishimoto, T., Hirano, J., Nishikata, S., Sawada, K., Kurokawa, S., et al. (2020). Neuronal network mechanisms associated with depressive symptom improvement following electroconvulsive therapy. *Psychol Med***,** 1-8. doi: 10.1017/S0033291720001518.

Takamiya, A., Kishimoto, T., Liang, K.C., Terasawa, Y., Nishikata, S., Tarumi, R., et al. (2019). Thalamic volume, resting-state activity, and their association with the efficacy of electroconvulsive therapy. *J Psychiatr Res* 117**,** 135-141. doi: 10.1016/j.jpsychires.2019.08.001.

Thomann, P.A., Wolf, R.C., Nolte, H.M., Hirjak, D., Hofer, S., Seidl, U., et al. (2017). Neuromodulation in response to electroconvulsive therapy in schizophrenia and major depression. *Brain Stimul* 10(3)**,** 637-644. doi: 10.1016/j.brs.2017.01.578.

van Waarde, J.A., Scholte, H.S., van Oudheusden, L.J.B., Verwey, B., Denys, D., and van Wingen, G.A. (2015). A functional MRI marker may predict the outcome of electroconvulsive therapy in severe and treatment-resistant depression. *Molecular Psychiatry* 20(5)**,** 609-614. doi: 10.1038/mp.2014.78.

Wang, J., Ji, Y., Li, X., He, Z., Wei, Q., Bai, T., et al. (2020a). Improved and residual functional abnormalities in major depressive disorder after electroconvulsive therapy. *Prog Neuropsychopharmacol Biol Psychiatry* 100**,** 109888. doi: 10.1016/j.pnpbp.2020.109888.

Wang, J., Wei, Q., Bai, T., Zhou, X., Sun, H., Becker, B., et al. (2017). Electroconvulsive therapy selectively enhanced feedforward connectivity from fusiform face area to amygdala in major depressive disorder. *Soc Cogn Affect Neurosci* 12(12)**,** 1983-1992. doi: 10.1093/scan/nsx100.

Wang, J., Wei, Q., Wang, L., Zhang, H., Bai, T., Cheng, L., et al. (2018a). Functional reorganization of intra- and internetwork connectivity in major depressive disorder after electroconvulsive therapy. *Hum Brain Mapp* 39(3)**,** 1403-1411. doi: 10.1002/hbm.23928.

Wang, J., Wei, Q., Yuan, X., Jiang, X., Xu, J., Zhou, X., et al. (2018b). Local functional connectivity density is closely associated with the response of electroconvulsive therapy in major depressive disorder. *J Affect Disord* 225**,** 658-664. doi: 10.1016/j.jad.2017.09.001.

Wang, L., Wei, Q., Wang, C., Xu, J., Wang, K., Tian, Y., et al. (2020b). Altered functional connectivity patterns of insular subregions in major depressive disorder after electroconvulsive therapy. *Brain Imaging Behav* 14(3)**,** 753-761. doi: 10.1007/s11682-018-0013-z.

Wei, Q., Bai, T., Brown, E.C., Xie, W., Chen, Y., Ji, G., et al. (2020a). Thalamocortical connectivity in electroconvulsive therapy for major depressive disorder. *J Affect Disord* 264**,** 163-171. doi: 10.1016/j.jad.2019.11.120.

Wei, Q., Bai, T., Chen, Y., Ji, G., Hu, X., Xie, W., et al. (2018). The Changes of Functional Connectivity Strength in Electroconvulsive Therapy for Depression: A Longitudinal Study. *Front Neurosci* 12**,** 661. doi: 10.3389/fnins.2018.00661.

Wei, Q., Ji, Y., Bai, T., Zu, M., Guo, Y., Mo, Y., et al. (2020b). Enhanced cerebro-cerebellar functional connectivity reverses cognitive impairment following electroconvulsive therapy in major depressive disorder. *Brain Imaging Behav*. doi: 10.1007/s11682-020-00290-x.

Wei, Q., Tian, Y., Yu, Y., Zhang, F., Hu, X., Dong, Y., et al. (2014). Modulation of interhemispheric functional coordination in electroconvulsive therapy for depression. *Translational Psychiatry* 4**,** e453. doi: 10.1038/tp.2014.101.

Wolf, R.C., Nolte, H.M., Hirjak, D., Hofer, S., Seidl, U., Depping, M.S., et al. (2016). Structural network changes in patients with major depression and schizophrenia treated with electroconvulsive therapy. *Eur Neuropsychopharmacol* 26(9)**,** 1465-1474. doi: 10.1016/j.euroneuro.2016.06.008.

Zhang, T., Bai, T., Xie, W., Wei, Q., Lv, H., Wang, A., et al. (2020). Abnormal connectivity of anterior-insular subdivisions and relationship with somatic symptom in depressive patients. *Brain Imaging Behav*. doi: 10.1007/s11682-020-00371-x.
